# Sestrin2 protects against lethal sepsis by suppressing the pyroptosis of dendritic cells

**DOI:** 10.1007/s00018-021-03970-z

**Published:** 2021-11-06

**Authors:** Li-xue Wang, Chao Ren, Ren-qi Yao, Yi-nan Luo, Yue Yin, Yao Wu, Ning Dong, Xiao-mei Zhu, Yong-ming Yao

**Affiliations:** 1 Chinese PLA General Hospital and Medical School of Chinese PLA, Beijing, 100853 People’s Republic of China; 2 Translational Medicine Research Center, Medical Innovation Research Division and Fourth Medical Center of the Chinese PLA General Hospital, Beijing 51 Fucheng Road, Haidian District, Beijing, 100048 People’s Republic of China; 3 Department of Burn Surgery, The First Affiliated Hospital of Naval Medical University, Shanghai, 200433 People’s Republic of China

**Keywords:** Pyroptosis, Sestrin2, Sepsis, Inflammasome, Endoplasmic reticulum stress, Dendritic cells

## Abstract

Sepsis is defined as life-threatening organ dysfunction caused by a dysregulated host response to infection. Sestrin2 (SESN2), a highly evolutionarily conserved protein, is critically involved in the cellular response to various stresses and has been confirmed to maintain the homeostasis of the internal environment. However, the potential effects of SESN2 in regulating dendritic cells (DCs) pyroptosis in the context of sepsis and the related mechanisms are poorly characterized. In this study, we found that SESN2 was capable of decreasing gasdermin D (GSDMD)-dependent pyroptosis of splenic DCs by inhibiting endoplasmic reticulum (ER) stress (ERS)-related nucleotide-binding oligomerization domain-like receptor protein 3 (NLRP3)-mediated ASC pyroptosome formation and caspase-1 (CASP-1) activation. Furthermore, SESN2 deficiency induced NLRP3/ASC/CASP-1-dependent pyroptosis and the production of proinflammatory cytokines by exacerbating the PERK–ATF4–CHOP signaling pathway, resulting in an increase in the mortality of septic mice, which was reversed by inhibiting ERS. These findings suggest that SESN2 appears to be essential for inhibiting NLRP3 inflammasome hyperactivation, reducing CASP-1-dependent pyroptosis, and improving sepsis outcomes through stabilization of the ER. The present study might have important implications for exploration of novel potential therapeutic targets for the treatment of sepsis complications.

**Supplementary Information:**

The online version contains supplementary material available at 10.1007/s00018-021-03970-z.

## Introduction

Sepsis is defined as life-threatening organ dysfunction caused by a dysregulated host response to infection and remains one of the predominant medical challenges in intensive care units [1, 2]. The degree to which the number of splenic leukocytes, including macrophages, dendritic cells (DCs), and T lymphocytes, is reduced is a critical determinant of the severity of sepsis and the prognosis of patients with sepsis and septic shock [3, 4]. DCs are the most important professional antigen-presenting cells and play pivotal roles in initiating adaptive immune responses [5, 6]. It has been documented that depletion of DCs occurs in the early stages of sepsis and that the extent of depletion is strongly associated with fatal outcomes among septic patients [7, 8]. However, the precise mechanisms underlying the depletion of DCs during sepsis remain unclear.

Pyroptosis is a subtype of programmed necrotic cell death that induces inflammation by promoting the activation of at least one type of cytosolic inflammasome [9]. Inflammasomes, which constitute a class of cytoplasmic multi-protein complexes, are programmed to selectively sense pathogen-associated molecular patterns (PAMPs) and danger-associated molecular patterns (DAMPs) [10, 11]. The nucleotide-binding oligomerization domain (NOD)-like receptor protein 3 (NLRP3) inflammasome is the most comprehensively characterized canonical inflammasome, and its activation can promote the activation of caspase-1 (CASP-1) via recruitment of the adaptor apoptosis speck-like protein containing a caspase recruitment domain (ASC) in response to various pathogenic, endogenous, and environmental danger signals [12, 13]. Gasdermin D (GSDMD), the critical executioner of pyroptotic cell death, is cleaved [14], and the GSDMD N-terminal domain (GSDMD-N) oligomerizes into a ring-like structure to form transmembrane pores, resulting in cell lysis and cytosolic contents release, which are the main characteristics of pyroptosis [15, 16]. It was previously reported that several signaling pathways, including endoplasmic reticulum (ER) stress (ERS), are involved in inflammasome activation [17]. The inflammasome is important for clearing invaded pathogens [18]; however, excessive activation of the inflammasome induces damage and dysfunction of non-infected tissue and multiple organs, leading to the development of inflammatory diseases [19]. Therefore, it is of great importance to explore the specific intracellular pathways associated with NLRP3 inflammasome-related pyroptosis during sepsis.

The ER is a large endomembrane compartment that serves as a platform for linking cellular stress and the inflammatory response [20, 21]. A wide range of insults can affect the protein-folding capacity of the ER. When a large number of misfolded proteins accumulate in the ER lumen, the unfolded protein response (UPR) is triggered, resulting in the restoration of ER homeostasis through a decrease in protein translation and promotion of protein degradation. However, regulated cell death is initiated when restoration of ER homeostasis by the UPR fails [22]. Our previous studies showed that prolonged ERS is responsible for apoptosis and dysfunction of DCs, which contribute to disruption of the host immune system in the context of both sepsis and severe thermal injury [23, 24]. Increasing studies have highlighted the pivotal role of ERS in inducing inflammasomes activation. As reported, in nonalcoholic steatohepatitis models, activation of the protein kinase RNA-like ER kinase (PERK)-activating transcription factor 4 (ATF4) pathway is capable of increasing C/EBP homologous protein (CHOP) expression, further enabling activation of the NLRP3 inflammasome followed by increased secretion of inflammatory cytokines, by promoting the pyroptosis of hepatocytes [25].

It has been demonstrated that three pathways involving ERS markers, i.e., PERK/ATF4, inositol-requiring enzyme 1 (IRE1)-X-box binding protein 1 (XBP1) and ATF6, are responsible for enhanced expression of Sestrin2 (SESN2) in infectious and inflammatory diseases [26]. The expression of SESN2, a stress-inducible protein, is upregulated in various immune cells in response to septic insults, and SESN2 plays protective roles under septic conditions by serving as an anti-inflammation, anti-apoptotic, and antioxidative target [26, 27]. Furthermore, an early study confirmed that SESN2 is capable of preventing persistent activation of the NLRP3 inflammasome. Deficiency of *SESN2*, however, results in overactivation of CASP-1 and excessive production of inflammatory cytokines, e.g., interleukin (IL)-1β and IL-18 [28]. Our previous report verified that SESN2 alleviates excessive inflammation and dysfunction of DCs by interacting with ATF4 to decrease the extent of ERS during sepsis [29]. Nevertheless, the potential role of SESN2 in NLRP3–CASP-1-dependent pyroptosis and its relationship with ERS remain unclarified. Therefore, the present study was carried out to investigate the protective impacts and mechanisms of SESN2 in regulating NLRP3–CASP-1-mediated pyroptosis of DCs in the context of septic challenge.

## Results

### Sepsis induces NLRP3–CASP-1-dependent pyroptosis of splenic DCs

**Fig. 1:**
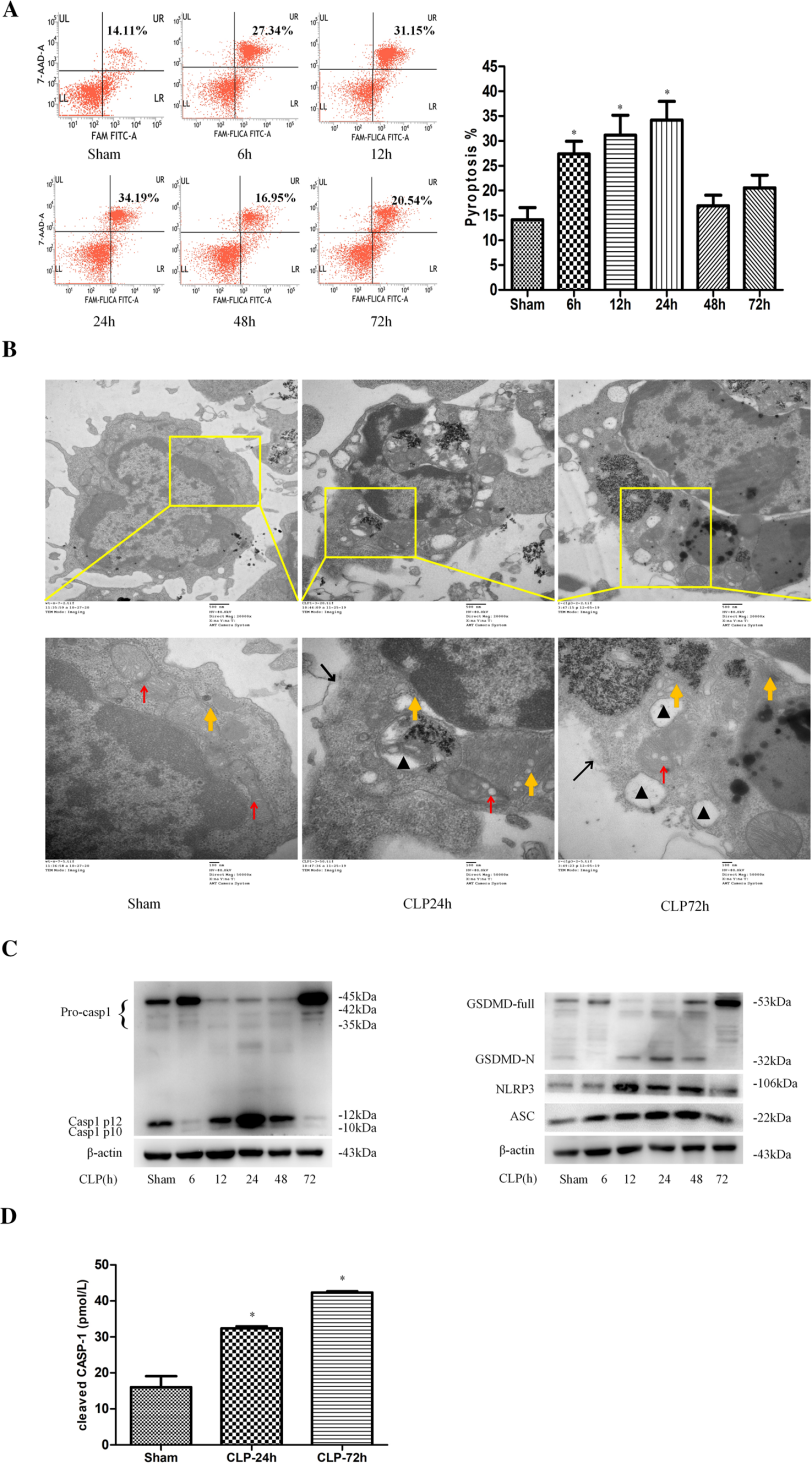
Sepsis induces pyroptosis and NLRP3 inflammasome activation in splenic DCs. DCs were isolated from spleens at 6 h, 12 h, 24 h, 48 h, and 72 h after CLP or sham operation. A, The DC pyroptosis was analyzed by flow cytometry. DCs were stained with CASP-1 (FAM-FLICA) and 7-AAD, and analyzed using two-color flow cytometry. Representative flow cytometry plots are shown on the left, and quantitative analysis of FLICA^+^ 7AAD^+^ DCs is shown on the right (*n* = 6). The data are presented as the mean ± SD. B, DC morphology was visualized by electron microscopy at 24 and 72 h after CLP. Representative electron micrographs (20,000 × , 50,000 ×) of morphological changes in the cell membrane, ER, and mitochondria of treated splenic DCs (the red arrows indicate mitochondria, the orange arrows indicate the ER, the black triangles indicate cytoplasmic vacuoles, and the black arrows indicate membrane pores). C, Immunoblotting was employed to measure the protein expression of NLRP3, CASP-1, ASC, and GSDMD. The values are protein levels relative to β-actin level. The data shown are representative of 3 independent experiments (3 mice were pooled together for each independent experiment). D, The plasma levels of cleaved CASP-1 were measured by ELISA. The data are expressed as the mean ± SD (*n* = 5). Sepsis was induced by CLP, and the sham group mice underwent surgery without CLP. Statistical significance: ^*^P < 0.05 versus the control group

Splenic DCs were harvested from mice at different time points after cecal ligation and puncture (CLP)-induced sepsis. We examined the DC pyroptosis rate and found that it was significantly increased at 6 h post CLP, and peaked at 24 h (Fig. 1A). Moreover, unlike DCs in the sham group, DCs in the CLP groups showed morphological features of pyroptosis, such as membrane collapse, cytoplasmic swelling, perinuclear chromatin aggregation, cytoplasmic vacuolization, and swelling of the ER and mitochondria at 24 h and 72 h after CLP (Fig. 1B).

The protein levels of NLRP3, ASC, cleaved CASP-1, and GSDMD-N were significantly increased at 12, 24, and 48 h after CLP compared with those in the sham group (Fig. 1C). We also measured the plasma levels of cleaved CASP-1 and noted that they were elevated at 24 h and 72 h after CLP (Fig. 1D). These results suggested that consistent with DC pyroptosis, the NLRP3 inflammasome was activated after sepsis. In accordance with the in vivo results, the expression of NLRP3, ASC, cleaved CASP-1, and GSDMD-N in DCs was significantly upregulated after priming with 1 μg/ml lipopolysaccharide (LPS) for 24 h and subsequent treatment with nigericin (Nig, an activator of the NLRP3 inflammasome) for 30 min in vitro (Fig. S1A). In addition, cleaved CASP-1 levels in the supernatants of stimulated DCs were identical to those found in cellular lysates by Western blotting (Fig. S1B).

### The proinflammatory microenvironment in the context of septic challenge

Given that the release of inflammatory cytokines, such as IL-1β and IL-18, is a hallmark of inflammasome activation and pyroptosis, we examined the secretion of IL-1β, IL-18, and other inflammatory cytokines both in vivo and in vitro. Plasma levels of IL-1β, IL-6, IL-18, and tumor necrosis factor (TNF)-α were elevated after CLP and peaked at 24 h, which was in line with the activation of NLRP3–CASP-1 inflammasome markers. High mobility group box-1 protein (HMGB1) expression was upregulated 6 h after CLP, while the IL-25 level was slightly higher at 24 h after CLP than in the sham group. However, the IL-10 level was increased at 24 h following septic challenge and peaked at 72 h (Fig. S2A. a–g). Additionally, treatment with LPS and Nig significantly increased the concentrations of these cytokines in the supernatants of DCs (Fig. S2B. a–g). Thus, these results indicated that the initiation of pyroptosis might be closely associated with the cytokine storm in the early phase of sepsis.

### Splenic DCs express NLRP3 inflammasome components during sepsis

**Fig. 2:**
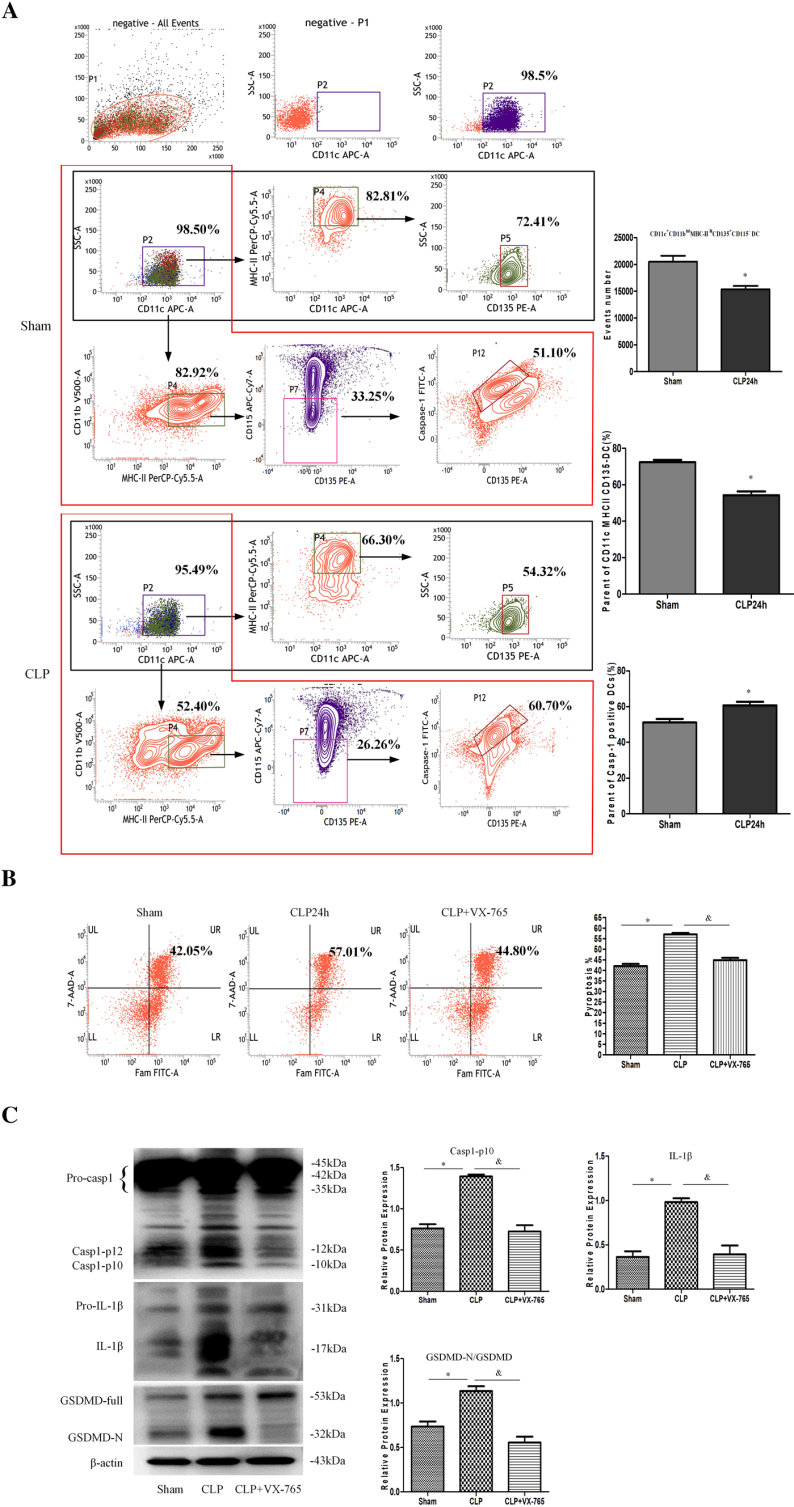
Splenic DCs express NLRP3 inflammasome components. Splenic DCs were isolated from mice at 24 h after CLP or sham operation. A, Representative flow cytometry plots showing the gating strategy used to distinguish DCs and macrophages using the markers of CD11c, CD11b, MHC-II, Flt3 (CD135), CD115. CASP-1 (FAM-FLICA) staining of primary splenic CD11c^+^CD11b^int^MHC-II^hi^CD135^+^CD115^−^ DCs was analyzed by flow cytometry. The numbers of CD11c^+^CD11b^int^MHC-II^hi^CD135^+^CD115^−^ DCs, the parents of CD11c^+^ MHC-II^hi^CD135^+^ DCs and the parents of CASP-1^+^ DCs are shown on the right. B, The DC pyroptosis rate was assessed by flow cytometry, and DCs were labeled with active CASP-1 (FAM-FLICA) and 7-AAD. Representative flow cytometry plots are shown on the left, and quantitative analysis of FLICA^+^ 7AAD^+^ DCs is shown on the right (*n* = 6). C, The protein expression levels of CASP-1, GSDMD, and IL-1β were determined by Western blotting. The values are protein levels relative to the β-actin level. The data shown are representative of 3 independent experiments. Statistical significance: ^*^P < 0.05 versus the sham or control group

A previous experiment reported that the expression of inflammasome components is heterogeneous in different subsets of DCs and that granulocyte–macrophage colony-stimulating factor-induced monocyte-like DCs do not express NLRP3–ASC–CASP-1 inflammasome components when treated with various stimuli, while primary splenic DCs exhibit an obvious inflammasome signature [30, 31]. In the current study, we isolated primary splenic CD11c^+^ DCs based on the expression of surface markers, including CD11b, major histocompatibility complex class II (MHC-II), Flt3 (CD135), and CD115. We found that the number and percentage of CD11c^+^MHC-II^hi^CD135^+^ DCs were decreased following sepsis (Fig. 2A), which is consistent with the results of previous studies [32]. CASP-1 activation was markedly enhanced in primary splenic CD11c^+^CD11b^int^MHC-II^hi^CD135^+^CD115^−^ DCs at 24 h after CLP (Fig. 2A). Moreover, the percentages of CASP-1-positive and TUNEL-positive cells were significantly increased, and the colocalization of NLRP3 and ASC was obviously increased at 24 h after CLP compared with those in the sham group (Fig. S3).

To further confirm the effect of CASP-1 inflammasome activation during sepsis, we examined changes in the DCs pyroptosis rates. The DC pyroptosis rates was significantly increased at 24 h post CLP but decreased by pretreatment with VX-765 (a selective inhibitor of CASP-1) (Fig. 2B). The expression of cleaved CASP-1, GSDMD-N, and IL-1β was markedly increased at 24 h following CLP, while pretreatment with VX-765 inhibited the activation of cleaved CASP-1, GSDMD-N, and IL-1β (Fig. 2C). Thus, suppression of CASP-1 activity can attenuate DC pyroptosis secondary to sepsis.

### ERS induces NLRP3/CASP-1-dependent pyroptosis and SESN2 expression in DCs

**Fig. 3:**
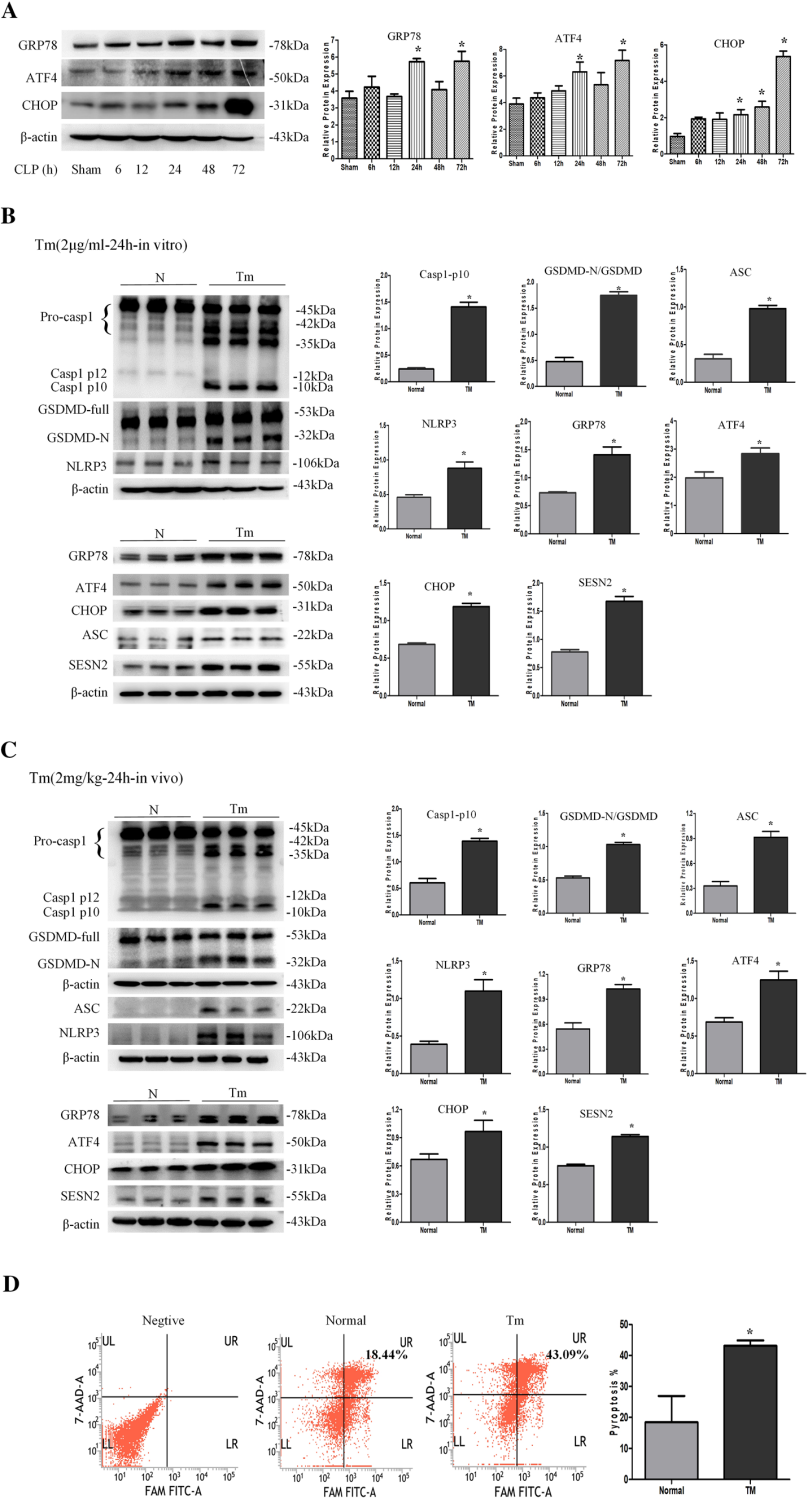
ERS induces NLRP3/CASP-1-dependent pyroptosis and SESN2 expression in DCs. A, Western blotting was used to measure the expression of ERS markers (GRP78, ATF4, and CHOP) in DCs at 6 h, 12 h, 24 h, 48 h, or 72 h post CLP. B, Primary DCs were treated with 2 μg/ml TM for 24 h, and Western blotting was used to evaluate the expression of SESN2, NLRP3 inflammasome components, and ERS markers. C, Assays were performed 24 h after the mice were intraperitoneally administered physiological saline or TM (2 mg/kg body weight). Immunoblots of SESN2, NLRP3 inflammasome components, and ERS markers are shown. The values are protein levels relative to the β-actin levels. The data are representative of 3 independent experiments (5 mice were pooled together for each independent experiment). D, DC pyroptosis was assessed by flow cytometry after treatment with 2 μg/ml TM for 24 h in vitro, DCs were labeled with active CASP-1 (FAM-FLICA) and 7-AAD. Representative flow cytometry plots are shown on the left, and quantitative analysis of FLICA^+^ 7AAD^+^ DCs is shown on the right (*n* = 4). Statistical significance: ^*^P < 0.05 versus the control group

To assess the activation of PERK–ATF4–CHOP signaling during sepsis, we examined the expression of glucose regulated-protein (GRP)78, ATF4, and CHOP at various time points during sepsis. The expressions of GRP78, ATF4, and CHOP was significantly enhanced in DCs from 24 to 72 h after CLP, and CHOP expression peaked at 72 h (Fig. 3A). Additionally, the protein levels of GRP78, ATF4, and CHOP were augmented after treatment with LPS following Nig administration (Fig. S4A).

To further investigate the relationship between ERS and pyroptosis, we used tunicamycin (TM) as a specific ERS inducer both in vivo and in vitro. According to our previous study, DCs were treated with 2 μg/ml TM for 24 h. Following TM treatment, the NLRP3 inflammasome was significantly activated and the expression of ASC, NLRP3 and cleaved CASP-1 was upregulated (Fig. 3B). Furthermore, GSDMD-N formation was increased upon NLRP3–CASP-1 inflammasome activation (Fig. 3B). Simultaneously, TM treatment obviously increased the expression of SESN2. In vivo, 2 mg/kg TM significantly elevated the expression of SESN2 and the activation of NLRP3, ACS, cleaved CASP-1, and GSDMD-N in DCs (Fig. 3C), and the DC pyroptosis rate was increased after TM exposure in vitro (Fig. 3D). Moreover, we analyzed the potential role of ERS in mediating DC pyroptosis. As shown in Fig. S4B, inhibition of ERS by pretreatment with salubrinal (a specific inhibitor of eIF2α dephosphorylation) for 1 h followed by treatment with LPS and Nig remarkably decreased NLRP3, cleaved CASP-1, and ASC activation and reduced SESN2 expression. These results suggested that ERS might be responsible for NLRP3 inflammasome-mediated pyroptosis and the expression of SESN2 during sepsis.

### The expression of SESN2 under septic condition

**Fig. 4:**
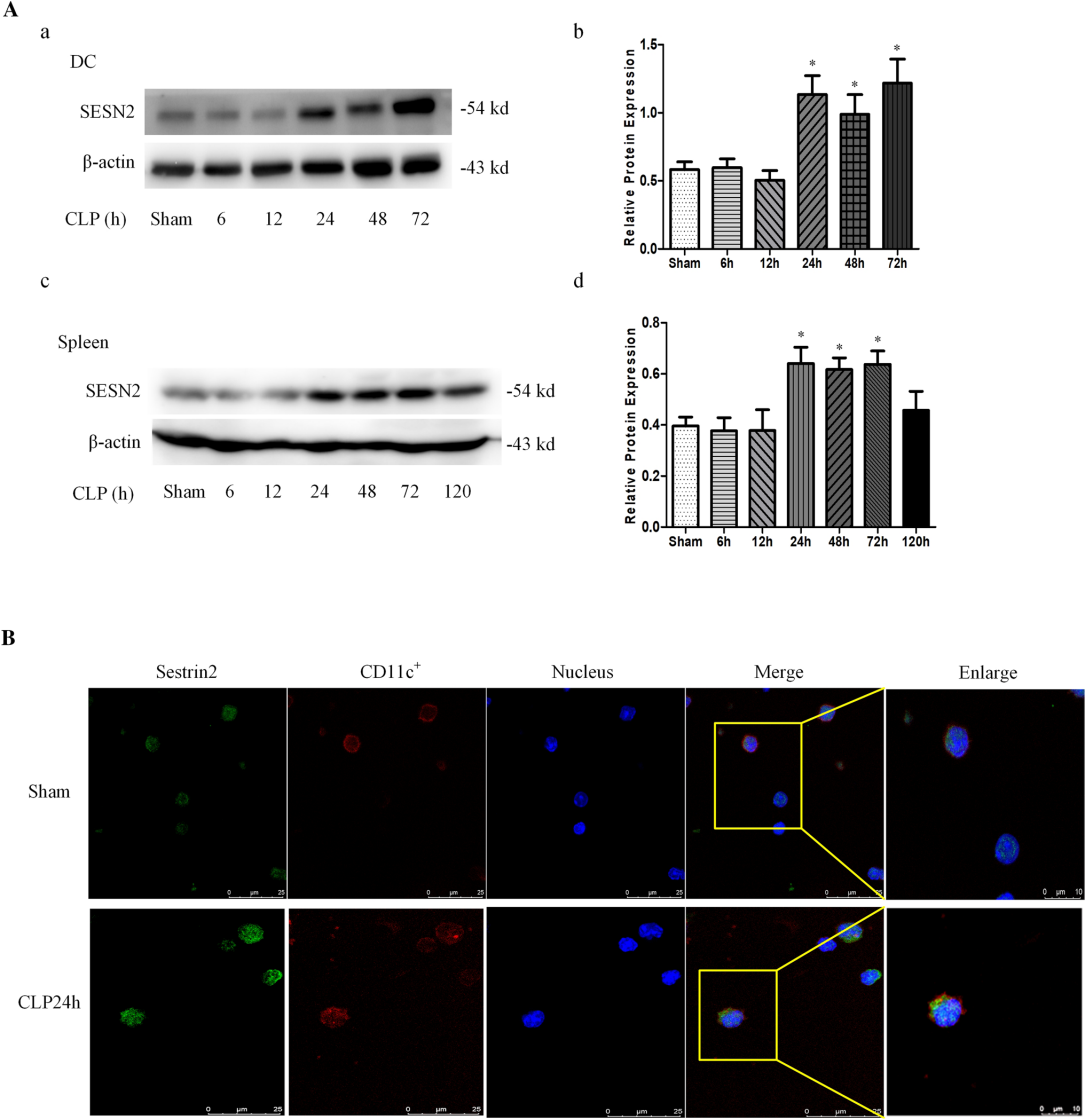
The expression of SESN2 in DCs and spleens under septic conditions. A, The protein expression of SESN2 in DCs and spleens after CLP (5 mice per group) was examined by Western blotting. A(**a**, **b**) SESN2 levels in DCs were examined at 6 h, 12 h, 24 h, 48 h, or 72 h after CLP. A (**c**, **d**) The expression of SESN2 in the spleen was analyzed at 6 h, 12 h, 24 h, 48 h, and 72 h, and on day 5 following the onset of sepsis. B, The protein expression of SESN2 in DCs was assessed by laser confocal scanning microscopy at 24 h after CLP. CD11c was labeled with APC, SESN2 was labeled with DyLight 488 (green), and nuclei were stained with DAPI (blue). The images are representative of six independent spleen samples from the CLP group. The data are presented as the mean ± SD of at least three independent experiments. Statistical significance: ^*^P < 0.05 versus the control group

To determine the protective impact of SESN2 against DC pyroptosis under septic conditions, we quantified the expression of SESN2 in both the spleens and splenic DCs of mice subjected to CLP or treated with LPS. In vivo, the protein level of SESN2 in splenic DCs was increased at 24 h after CLP and peaked at 72 h compared with that in the sham group (Fig. 4A a, b). Consistently, the protein expression of SESN2 in the spleen was significantly upregulated at 24 h but decreased on day 5 after CLP (Fig. 4A c, d). Moreover, the fluorescence intensity of SESN2 in CD11c^+^ DCs was markedly enhanced at 24 h after CLP (Fig. 4B). In vitro, treatment with LPS followed by Nig also upregulated SESN2 expression in DCs in a time-dependent manner (Fig. S5).

### *SESN2*^−/−^ DCs exhibit severe ERS response, pyroptosis, and inflammatory damage during sepsis

#### SESN2 deficiency exacerbates sepsis-induced ERS

**Fig. 5:**
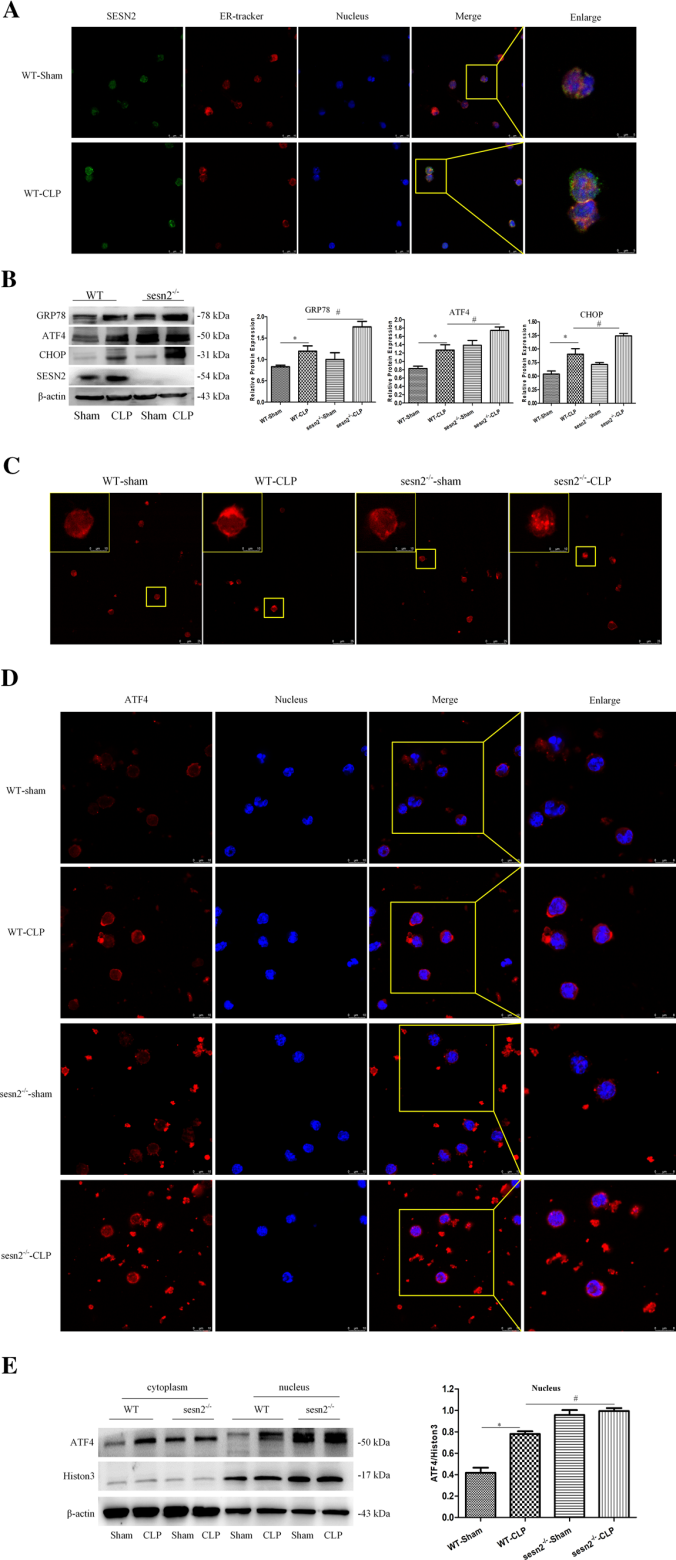
SESN2 deficiency exacerbates sepsis-induced ERS. WT mice and SESN2^−/−^ mice were subjected to sham surgery or CLP. A, SESN2 expression and the ER in DCs were examined by laser scanning confocal microscopy 24 h after CLP. DyLight 488 (green)-labeled SESN2 protein, ER tracker (red), and DAPI-stained nuclei (blue) were visualized using indirect immunofluorescence and confocal microscopy (× 600, × 1200). B, Immunoblot analysis of GRP78, ATF4, and CHOP expression in DCs from WT and SESN2^−/−^ mice at 24 h after CLP. C, Morphological alterations in the ER in DCs from WT and SESN2^−/−^ mice were measured by immunofluorescence staining at 24 h after CLP (× 600, × 1200). D, ATF4 (DyLight 594, red) expression in DCs was observed by using indirect immunofluorescence and confocal microscopy, and DAPI-stained nuclei (blue) in DCs from WT and SESN2^−/−^ mice were visualized at 24 h after CLP (× 600, × 1200). E, Immunoblot analysis of ATF4 expression in the cytosol and nucleus. β-actin served as a control for cytosolic proteins. Histone3 served as a control for nuclear proteins. The data are presented as the mean ± SD of at least three independent experiments. Statistical significance: ^*^P < 0.05 versus the sham group

In line with our previous study [29], SESN2 was colocalized with ER, and the fluorescence intensity of SESN2 was enhanced at 24 h after CLP (Fig. 5A). As shown in Fig. 5B, SESN2 deficiency significantly exacerbated ERS at 24 h after CLP, as indicated by upregulated expression of GRP78, ATF4, and CHOP compared with that in the wild-type (WT) group. Moreover, GRP78, ATF4, and CHOP protein levels were increased in SESN2^−/−^ DCs after priming with LPS and subsequent treatment with Nig (Fig. S6). Additionally, expansion and fragmentation of the ER were aggravated (Fig. 5C) and ATF4 activity and nuclear location were obviously increased in the SESN2 knockout group compared with the WT group at 24 h after CLP (Fig. 5D). Immunoblotting indicated that SESN2 knockout facilitated the translocation of ATF4 to the nucleus following septic challenge, in turn causing excessive ERS (Fig. 5E).

#### SESN2 deficiency exacerbates pyroptosis and hyperactivation of the NLRP3 inflammasome

**Fig. 6:**
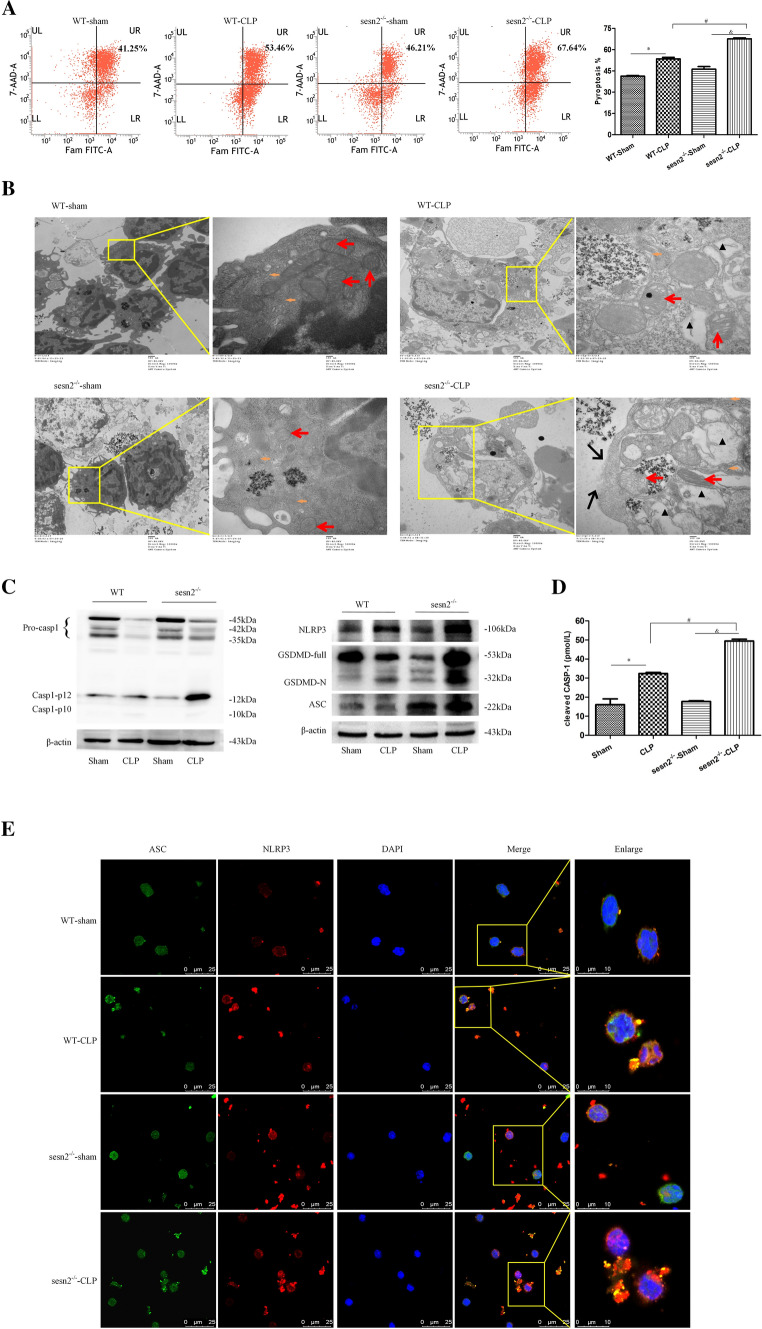
Genetic deficiency of SESN2 exacerbates pyroptosis and NLRP3 inflammasome hyperactivation in sepsis. DCs were isolated from the spleen at 24 h following CLP. A, Pyroptosis of DCs was analyzed by flow cytometry, and DCs were labeled with active CASP-1 (FAM-FLICA) and 7-AAD. Representative flow cytometry plots are shown on the left, and quantitative analyses of FLICA^+^ 7AAD^+^ DCs are shown on the right (*n* = 6). B, Representative electron micrographs (10,000 × , 50,000 ×) of morphological alterations in the cell membrane, cytoplasm, ER, and mitochondrial organelles of splenic DCs (the red arrows indicate mitochondria, the orange arrows indicate the ER, the black triangles indicate cytoplasmic vacuoles, and the black arrows indicate membrane pores). C, Immunoblot analysis of CASP-1, GSDMD, NLRP3, and ASC expression in DCs from WT and SESN2^−/−^ mice at 24 h after CLP. β-actin served as an internal control. D, The plasma levels of cleaved CASP-1 were measured by ELISA at 24 h after CLP (*n* = 5). E, Representative confocal immunofluorescence microscopy images of NLRP3 colocalized with ASC. DyLight 488 (green)-labeled ASC protein, DyLight 594 (red)-labeled NLRP3 protein, and DAPI (blue)-stained nuclei are shown. The images are representative of six independent spleen samples from the CLP group. The data are presented as the mean ± SD of at least three independent experiments. Statistical significance: ^*^P < 0.05 versus the control group

To investigate the effect of SESN2 in primary splenic mononuclear cells, we examined the number of CD11c^+^ cells among splenic lymphocytes. Sepsis induced a decrease in the number of CD11c^+^ splenic cells, while SESN2^−/−^ mice showed a significantly fewer CD11c^+^ splenic cells than WT mice at 24 h after CLP (Fig. S7A). In addition, DCs from SESN2^−/−^ mice showed significantly increased pyroptosis compared with those from WT mice at 24 h after CLP (Fig. 6A). Moreover, the CCK-8 assay showed that the viability of DCs was decreased by more 20% in the SESN2-deficient group compared to the WT group at 24 h after CLP (Fig. S7B). Electron microscopy revealed that DCs from WT and SESN2^−/−^ mice subjected to sham surgery displayed normal morphology; however, more severe rupture of the plasma membrane, greater cytoplasmic vacuolization, and more organelle swelling were observed in DCs from SESN2^−/−^ mice than those from WT mice at 24 h after CLP (Fig. 6B).

At 24 h after the onset of septic challenge, the activation of CASP-1, NLRP3, and ASC was aggravated and the processing of pro-GSDMD into GSDMD-N was significantly increased in the SESN2 knockout group (Fig. 6C). The plasma levels of cleaved CASP-1 were markedly elevated in SESN2^−/−^ mice subjected to CLP compared with those in WT mice (Fig. 6D). Similar to what was observed in the in vivo study, SESN2 knockout significantly increased the expressions of cleaved CASP-1, NLRP3, ASC, and GSDMD-N after LPS treatment followed by Nig administration (Fig. S7C). Much higher levels of cleaved CASP-1 were observed in the supernatant of SESN2^−/−^ DCs than in that of WT DCs after treatment with LPS and Nig (Fig. S7D). The colocalization of the NLRP3 and ASC proteins was higher in the sepsis group than in the sham group and was markedly increased in the SESN2^−/−^ septic mice compared to WT septic mice (Fig. 6E). These results indicated that SESN2 deficiency decreases the number of CD11c^+^ splenic cells and exacerbates DC pyroptosis and inflammasome hyperactivation during sepsis.

#### Knockout of SESN2 worsens the inflammatory response and septic outcomes in mice

**Fig. 7:**
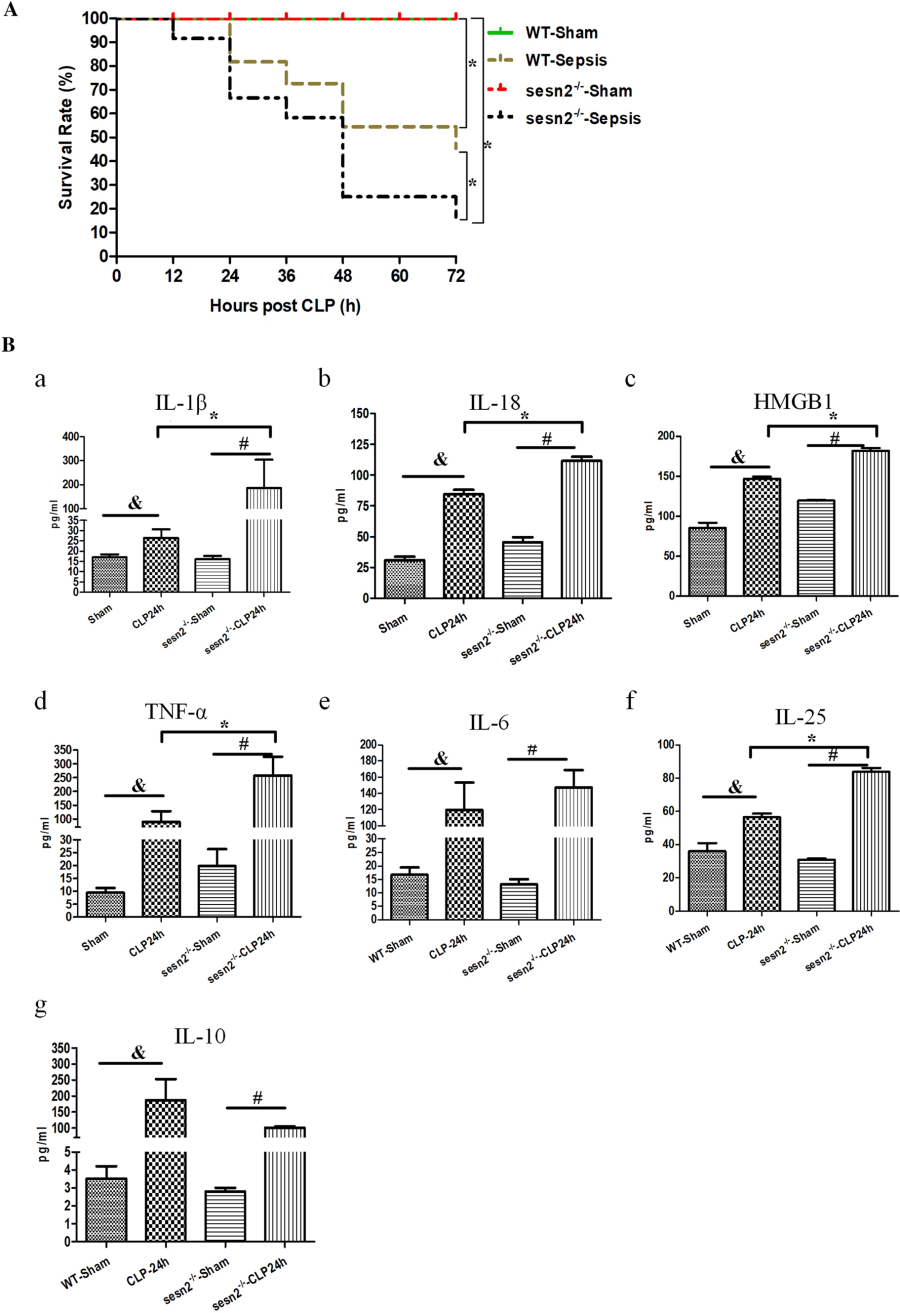
The effect of SESN2 on the inflammatory response and outcomes in septic mice. A, The survival rates of WT mice (*n* = 11) and SESN2^−/−^ mice (*n* = 12) were monitored at the indicated time points. B, The plasma levels of IL-1β, IL-6, IL-10, IL-18, IL-25, HMGB1, and TNF-α were measured by ELISA at 24 h after CLP (*n* = 7–9). The data are presented as the mean ± SD. Sepsis was induced by CLP in WT mice and SESN2^−/−^ mice. Mice in the sham group underwent surgery without CLP. Statistical significance: ^*^P < 0.05 versus the control group

To explore the potential role of SESN2 in the development of sepsis in vivo, we observed the survival rates of WT and SESN2^−/−^ mice after CLP. Compared to WT mice, SESN2^−/−^ mice exhibited significantly decreased survival rates (Fig. 7A). Overactivation of the inflammasome has been shown to exacerbate cell injury and inflammation during sepsis [15]. To test the effect of SESN2 on the inflammatory response in the context of sepsis, we measured the levels of inflammatory cytokines in both WT and SESN2^−/−^ mice. Consistent with the higher mortality rate observed in SESN2^−/−^ mice than in WT mice, the plasma levels of IL-1β, IL-6, IL-18, IL-25, HMGB1, and TNF-α were significantly elevated in SESN2^−/−^ mice compared with WT mice at 24 h after CLP (Fig. 7Ba–f). Although the expression of IL-10 was upregulated in the SESN2^−/−^ CLP group, the degree of the increase in IL-10 expression was less than that in the WT CLP group (Fig. 7Bg). In addition, very high concentrations of inflammatory cytokines (IL-1β, IL-6, IL-18, HMGB1, and TNF-α) (Fig. S8a–e) and a slight increase in IL-10 expression were observed in the supernatants of SESN2^−/−^ DCs after treatment with LPS followed by Nig (Fig. S8g). However, the level of IL-25 was unaffected (Fig. S8f). Taken together, these data indicated that SESN2 deficiency aggravated the inflammatory storm in the early stage of sepsis.

### The PERK–ATF4–CHOP pathway is critically involved in the inhibitory effects of SESN2 on pyroptosis

**Fig. 8:**
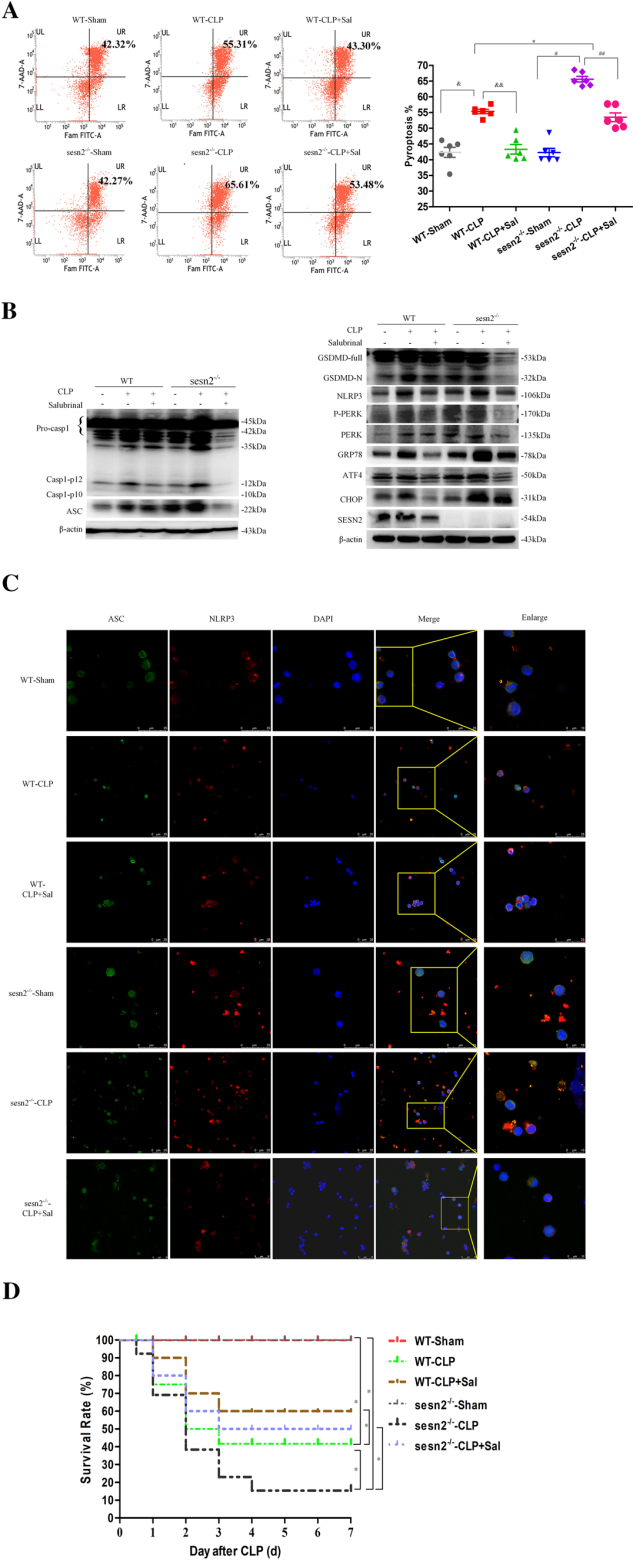
The PERK–ATF4–CHOP pathway is critically involved in the inhibitory effect of SESN2 on DC pyroptosis. DCs were isolated from the spleens of WT and SESN2^−/−^ mice treated with or without salubrinal at 24 h after sham surgery or CLP. A, DC pyroptosis was assessed by flow cytometry, and DCs were labeled with active CASP-1 (FAM-FLICA) and 7-AAD. Representative flow cytometry plots are shown on the left, and quantitative analyses of FLICA^+^ 7AAD^+^ DCs are shown on the right (*n* = 6). B, Immunoblot analysis of the expression of inflammasome markers, including CASP-1, GSDMD, NLRP3, and ASC and signaling molecules, including PERK, ATF4, CHOP, and GRP78. β-actin served as an internal control. C, Representative confocal immunofluorescence microscopy images of NLRP3 colocalized with ASC. DyLight 488 (green)-labeled ASC protein, DyLight 594 (red)-labeled NLRP3 protein, and DAPI (blue)-stained nuclei are shown. The images are representative of six independent samples from each group. The data are presented as the mean ± SD of at least three independent experiments. Statistical significance: ^*^P < 0.05 versus the control group. D, Kaplan–Meier survival curve analysis was performed for mice with CLP-induced sepsis for 7 days (n = 12). Mice in the sham group underwent surgery without CLP and received 4 ml/kg PBS at 1 h after the operation, mice in the CLP group received 4 ml/kg PBS at 1 h after CLP, and mice in the CLP + salubrinal group received 20 mg/kg salubrinal at 1 h after CLP

As shown in Fig. 8A, SESN2^−/−^ mice exhibited a marked increase in DC pyroptosis at 24 h after sepsis, which was reversed by pretreatment with salubrinal. Moreover, SESN2 deficiency aggravated the activation of NLRP3, cleaved CASP-1, and GSDMD-N, which is in line with the upregulation of the expression of ERS markers, including GRP78, phosphorylated (p)-PERK, ATF4, and CHOP (Figs. 8B, S9). Pretreatment with salubrinal prevented ERS from activating the PERK–ATF4–CHOP signaling pathway, and inhibited the effect of SESN2 on NLRP3 inflammasome activation (Figs. 8B, S9). The colocalization of the NLRP3 and ASC proteins was increased in SESN2^−/−^ DCs at 24 h after CLP, while pretreatment with salubrinal significantly decreased the activation of the NLRP3 inflammasome, as evidenced by the reduction in the colocalization of the NLRP3 and ASC proteins (Fig. 8C). Overall, these results revealed that SESN2 suppresses CHOP-mediated activation of NLRP3/CASP-1-dependent pyroptosis during sepsis.

### Impact of SESN2 on the survival rates of sepsis model mice

To evaluate the impact of SESN2 in sepsis and the molecular mechanism, we further examined the 7-day survival rates of mice subjected to CLP and pretreated with or without salubrinal. Sepsis mortality rates were markedly higher for SESN2^−/−^ mice then for WT mice, while treatment with salubrinal (2 mg/kg) 1 h prior to CLP provided significant protection against CLP-induced mortality in both WT and SESN2^−/−^ mice (Fig. 8D). Salubrinal significantly reduced the mortality rate of SESN2^−/−^ mice but not that of WT mice (Fig. 8D), which confirmed that SESN2 plays a protective role in sepsis by alleviating ERS. Therefore, SESN2 might inhibit excessive activation of PERK–ATF4–CHOP to suppress NLRP3/CASP-1 pathway-mediated pyroptosis of DCs following septic challenge.

## Discussion

Sepsis is a complex but severe immune disorder in response to various pathogens and is characterized by both excessive systemic inflammation and overwhelming immunosuppression [34]. Increased programmed cell death, such as apoptosis, pyroptosis or necroptosis, of immune cells contributes to a significant decrease in the number of effective immune cells in the circulation, resulting in host immune dysfunction during sepsis [35, 36]. The duration and extent of immune cell death are closely associated with the severity and mortality of septic patients [37]. Currently, it is widely accepted that the number of DCs is decreased and that DC dysfunction occurs due to the aberrant response of the immune system [38, 39]. Indeed, maintaining the activity of DCs has been shown to alleviate endotoxin-induced immune depression and mortality in septic mice [40].

Emerging evidence suggests that sepsis causes prolonged activation of the NLRP3 inflammasome, thereby contributing to a persistent inflammatory response and extensive cell injury, especially in innate immune cells [41]. However, Erlich et al. [30] showed that bone marrow-derived DCs comprised a heterogeneous population of DCs (CD11c^+^CD11b^int^MHC-II^hi^CD115^−^Flt3^+^) and monocyte-derived macrophages (CD11c^+^CD11b^hi^MHC-II^low^CD115^+^Flt3^−^) and highlighted the obvious difference between these cell subsets in inflammasome component expression and activation. It was also revealed that unliked bone marrow-derived DCs, primary splenic DCs express all inflammasome components. In a previous study, primary splenic DCs originating from common DC progenitors were isolated according to the expression of surface molecules, including CD11c, MHC-II, and Flt3 [42]. Flt3 is specifically expressed by DCs and serves as an excellent marker for distinguishing DCs from macrophages. Although macrophages can express low levels of CD11c and MHC-II, they express high levels of CD11b and CD115. Therefore, we distinguished CD11c^+^CD11b^hi^MHC-II^low^CD115^+^Flt3^−^ macrophages from CD11c^+^CD11b^int^MHC-II^hi^CD115^−^Flt3^+^ DCs on the basis of their phenotypic features and further compared pyroptosis of these cells. In the current study, activation of the NLRP3 inflammasome accompanied by increased secretion of IL-1β, IL-18, and HMGB1 was noted in CD11c^+^CD11b^int^MHC-II^hi^CD115^−^Flt3^+^ splenic DCs after induction of sepsis. Our results were in accordance with Erlich and Guermonprez, who showed that the splenic DC subset express of essential inflammasome components [30, 31]. Notably, our experiments revealed that the DC pyroptosis rate was significantly increased in response to septic insults, possibly due to a profound decrease in the number of these DCs. Collectively, these data suggest that pyroptosis has a cell type-dependent effect on the decrease in the number of splenic DCs during sepsis.

The NLRP3 inflammasome has been confirmed to be activated in monocytes and macrophages under septic conditions [43, 44]. Similarly, we found that significant activation of NLRP3–ASC–CASP-1-mediated pyroptosis and excessive release of inflammatory cytokines are mainly characteristics of the initial hyperinflammatory phase of sepsis. These data demonstrated that a complex yet dynamic immune/inflammatory process occurs during sepsis progression, hinting that treatments with both anti-inflammation and immunomodulatory effects are needed to improve sepsis outcomes. Interestingly, we found that the DC pyroptosis rate was decreased at 72 h after CLP, at which point the levels of cleaved CASP-1 in plasma and supernatant were still high. Our previous study showed that sepsis induces apoptosis of DCs in a time-dependent manner [29]. Thus, cleaved CASP-1 contributes to apoptosis induction, which might explain the inhibition of NLRP3–ASC–CASP-1-mediated pyroptosis. Additionally, several studies have noted a link between pyroptosis and apoptosis after infection [45, 46]. As reported, RNA virus-induced NLRP3 inflammasome activation can trigger the activation of CASP-3/7-dependent apoptosis [47]. Thus, we infer that pyroptosis and apoptosis are tightly connected and can cross-regulate each other; however, the mechanism underlying the switch between these two cell death types needs further study.

Growing evidence recently indicated that ERS is critically involved in the process of sepsis [33, 48]. Many studies have demonstrated that three ERS signaling pathways (the PERK/ATF4, IRE1/XBP1, and ATF6 pathways) can lead to CHOP transcription. Severe ERS, especially ERS via the PERK/ATF4/CHOP pathway, causes cell death under certain physiological and pathophysiological conditions. CHOP can serve as an important mediator of the inflammatory response in the development of sepsis [49]. Our findings indicated that the expression of ERS components (GRP78, ATF4, and CHOP) was significantly upregulated in DCs after the initiation of sepsis, showing the same tend as inflammasome activation, hinting at a relationship between PERK–ATF4–CHOP signaling and CASP-1-mediated pyroptosis. We further observed that an ER stressor (TM) significantly triggered NLRP3/CASP-1 activation and increased pyroptosis-related gene expression and the DC pyroptotic rate. However, inhibition of ATF4 signaling substantially decreased the expression of NLRP3 inflammasome components. Other studies have demonstrated that the NLRP3 inflammasome is activated during ERS, as its activation requires Ca^2+^, reactive oxygen species or potassium efflux [50, 51]. Nevertheless, the specific mechanism underlying ERS-induced NLRP3 inflammasome-dependent pyroptosis in sepsis remains to be elucidated.

The expression of SESN2, which acts as an essential molecule for maintaining homeostasis of the ER by decreasing ERS severity and exerts protective effects against ERS-associated diseases, was markedly increased in response to the activation of any of the three branches of the ERS response [52, 53]. Consistent with our previous study [29], SESN2 expression was significantly upregulated upon septic challenge, and SESN2 deficiency aggravated ER protein loading, subsequently leading to excessive ERS. Moreover, SESN2-deficient mice exhibited hyperactivation of the NLRP3 inflammasome and excessive production of inflammatory cytokines, which contributed to augmenting pyroptotic cell death and increasing the mortality of septic animals. The protective impact of SESN2 in regulating NLRP3 inflammasome-dependent pyroptosis is consistent with a recent report showing that SESN2 protects the host from sepsis by suppressing prolonged activation of the NLRP3 inflammasome [28]. Therefore, the present study advances our understanding of the inhibitory effect of SESN2 on the regulation of pyroptosis during ERS.

Although there is no evidence of the role of ERS in SESN2-induced modulation of NLRP3 inflammasome-mediated pyroptosis in sepsis, the link between ERS and NLRP3 is supported by findings from other models. For instance, Han et al. [54] found that CHOP is capable of interacting with the NLRP3 gene and is necessary for NLRP3 inflammasome activation in liver injury models. Inhibition of the PERK–CHOP pathway suppresses NLRP3 activation, which greatly attenuates ERS-induced hepatocyte injury. Of note, we further observed that inhibition of the PERK–ATF4–CHOP axis by salubrinal prevented SESN2 deficiency-induced excessive activation of the NLRP3 inflammasome and pyroptosis in DCs under septic conditions. Similarly, adiponectin prevents the development of breast cancer by inhibiting the NLRP3 inflammasome and ERS activation via the SESN2/AMPK axis [55]. These observations support the notion that SESN2 can serve as a key regulator for inhibiting ERS-associated pyroptosis by suppressing inflammasome activation secondary to sepsis.

Nonetheless, our study has some limitations. First, we verified the potential effects of SESN2 in regulating NLRP3/CASP-1-dependent pyroptosis through PERK–ATF4–CHOP signaling, but the precise mechanism underlying PERK–ATF4–CHOP signaling should be investigated in future experiments. However, we noticed that the noncanonical CASP-11 pathway is activated during sepsis, but we did not valuate the possible role of SESN2 in this noncanonical pathway, which is worth investigating in further study. Second, the potential effects of SESN2 and ATF4 double knockdown in sepsis models need to be further tested. Third, clinical studies are warranted in the near future to strengthen our observations and make reasonable clinical interpretations.

**Fig. 9:**
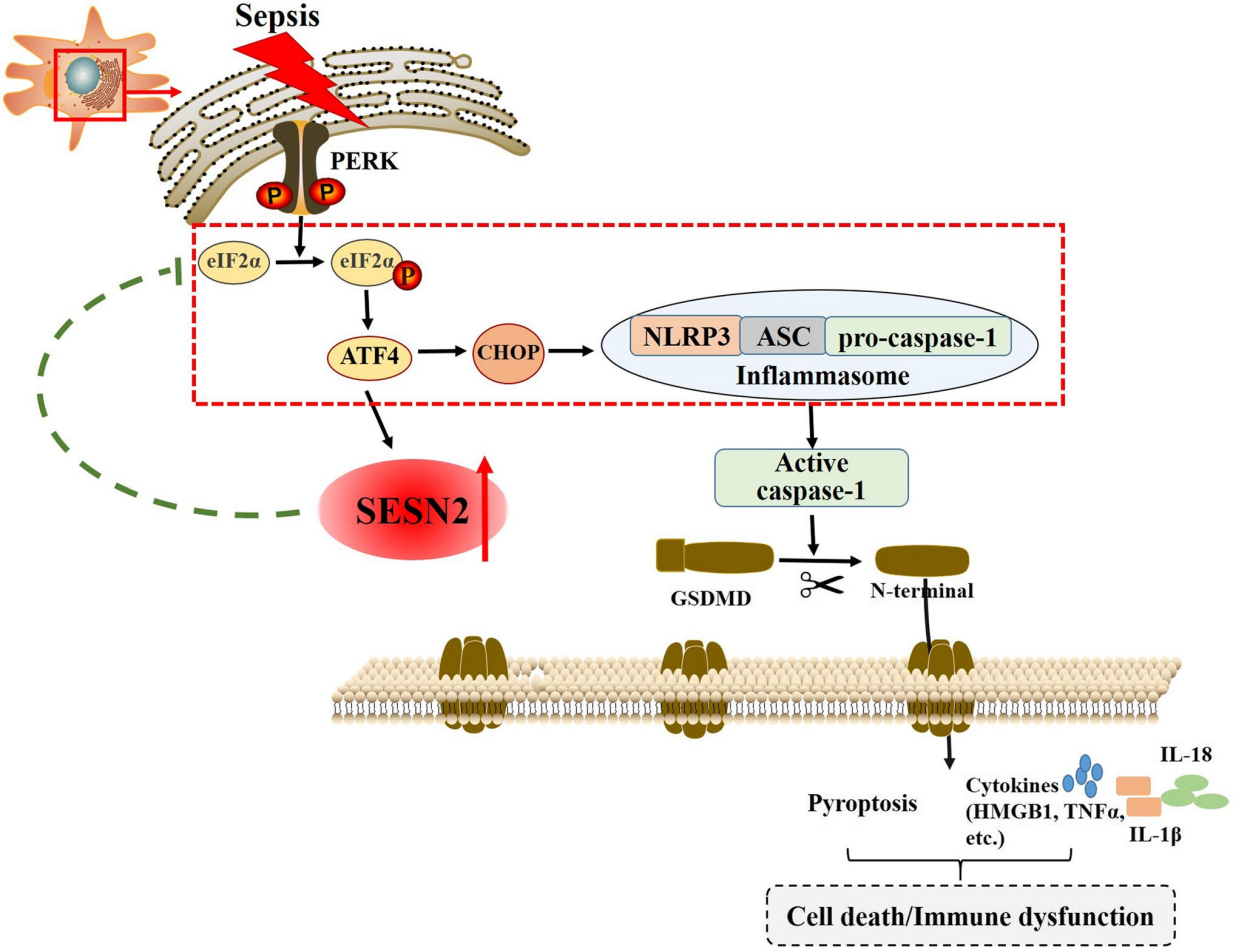
Basic schematic of the mechanism by which SESN2 inhibits pyroptosis and prolongs NLRP3 inflammasome activation by preserving ERS homeostasis. In splenic DCs, SESN2 expression was enhanced after CLP or stimulation with LPS via the PERK–ATF4–CHOP pathway. Sepsis per se induced severe latent ERS, and hyperactivation of ATF4 resulted in obvious elevation of CHOP expression, which might act as a potentiating step. Furthermore, CHOP activated the NLRP3 inflammasome and promoted the activation of pro-CASP-1 to CASP-1. Upon activation, CASP-1 cleaved the N-terminal fragment of GSDMD, and then GSDMD-N oligomerized in the plasma membrane and formed pores, resulting in the release of cellular contents and pyroptosis. Moreover, CASP-1 activation led to the maturation of IL-1β and IL-18 and the release of cytokines (IL-6, HMGB1, TNF-α, etc.), thereby contributing to the inflammatory response, immune dysfunction, and even cell death. Upregulation of SESN2 might reduce protein translation, and attenuate ERS, and subsequently suppress NLRP3 inflammasome activation through the PERK–ATF4–CHOP pathway, controlling the excessive inflammatory response and pyroptosis secondary to septic challenge

In summary, we propose that SESN2 impedes NLRP3–ASC–CASP-1-mediated pyroptosis by preserving ER homeostasis during sepsis. The expression of SESN2 significantly increases in response to ERS, which is triggered by both specific stimuli and sepsis. In turn, SESN2 might have a negative feedback effect on ERS, as genetic deletion of SESN2 leads to overactivation of the PERK–ATF4–CHOP pathway, thereby contributing to augmentation of NLRP3–ASC–CASP-1-mediated pyroptosis, uncontrolled inflammation, and increased mortality in septic animals. However, inhibition of ERS obviously abates the above-mentioned effects in the context of sepsis (Fig. 9). This study might have important implications for the exploration of novel potential therapeutic targets for the treatment of sepsis complications.

## Materials and methods

### Mice

WT C57BL/6 J mice (6–8 weeks old, male, weight range: 20–25 g) were purchased from the Institute of Laboratory Animal Science, Peking Union Medical College (Beijing, China). Sesn2^−/−^ mice on the C57BL/6 J background were generated by Shanghai Model Biological Center (Shanghai, China). The mice were housed under SPF conditions on a 12-h light/dark cycle for at least 3 days before the experiments. All experimental manipulations were conducted in accordance with the National Institutes of Health Guide for the Care and Use of Laboratory Animals with the approval of the Scientific Investigation Board of the Chinese PLA General Hospital, Beijing, China. WT and Sesn2^−/−^ mice were randomly divided into six groups: the WT sham group, the WT CLP group, the WT CLP + salubrinal group, the Sesn2^−/−^ sham group, the Sesn2^−/−^ CLP group, and the Sesn2^−/−^ CLP + salubrinal group.

### Reagents

CD11c^+^ (N418) microbeads were purchased from Miltenyi Biological GmbH (BergischGladbach, Germany). LPS (*E.coli* O127:B8) and TM were purchased from Sigma Aldrich (St. Louis, MO). A FAM-FLICA® CASP-1 assay kit was purchased from ImmunoChemistry Technologies (Bloomington, MN). Annexin-V-phycoerythrin (PE) and 7-AAD apoptosis detection kits were purchased from BD (San Diego, CA). An In Situ Cell Death Detection Kit, TMR Red was purchased from Roche Applied Science (Indianapolis, IN). Rabbit anti-mouse SESN2, rabbit anti-mouse CASP-1, rabbit anti-mouse GSDMD, rabbit anti-mouse GRP78, rabbit anti-mouse ATF4, and mouse anti-mouse DDIT3 monoclonal antibodies were purchased from Abcam (Cambridge, MA). Rabbit anti-mouse ASC/TMSA (D2W8U), rabbit anti-mouse PERK and rabbit anti-mouse p-PERK monoclonal antibodies were purchased from Cell Signaling Technology (Danvers, MA). A mouse anti-mouse NLRP3/NALP3 monoclonal antibody was purchased from AdipoGen (San Diego, CA), and a mouse anti-mouse ATF4 monoclonal antibody was purchased from Proteintech Group (Rosemont, IL). ER-Tracker red was purchased from Invitrogen (California, CA), and Alexa Fluor 488-conjugated goat anti-rabbit IgG (H + L) and Alexa Fluor 594-conjugated goat anti-mouse IgG (H + L) were purchased from Santa Cruz Biotechnology (Santa Cruz, CA). Triton X-100 was purchased from Sigma (St. Louis, MO), and salubrinal and VX-765 were purchased from Selleckchem (Houston, TX).

### Mouse model of CLP-induced sepsis

WT C57BL/6 J mice and Sesn2^−/−^ mice underwent CLP-induced sepsis. Laparotomy was performed to isolate the cecum after anesthesia (5% chloral hydrate, 400 mg/kg, i.p.). A specified percentage (2/3) of the cecum was ligated and punctured twice with a 21-gauge needle, and then the cecum was punctured to induce sepsis. Next, the cecum was returned to abdominal cavity. The abdominal wall and incision were closed with 6–0 silk sutures. After surgery, 1 ml of 0.9% normal saline was injected subcutaneously. Lethargy, diarrhea, and piloerection in the first 6 h after the operation indicated the successful establishment of the sepsis model. Mice in the sham group underwent the same surgical procedure without the ligation and puncture of the cecum.

### Isolation of splenic DCs

Under aseptic conditions, the mouse spleens were obtained and washed twice with precooled phosphate-buffered saline (PBS). Mononuclear cells were isolated, and splenic DCs were isolated from murine mononuclear cells using a CD11c^+^ DC isolation kit (MiltenyiBiotec, BergischGladbach, Germany) with a positive selection MS column following the manufacturer’s instructions. The selected DCs were obtained by centrifugation at 200×*g* for 10 min, and the supernatant was discarded. Then, these isolated cells were cultured in Roswell Park Memorial Institute (RPMI) 1640 medium containing 10% heat-inactivated fetal bovine serum (FBS), 100 U/ml penicillin and 100 μl/ml streptomycin at 5% CO_2_ and 37 ˚C in a humidified incubator, or used for experiments.

### Cell culture and stimulation

DCs were cultured in RPMI 1640 medium containing 10% heat-inactivated FBS, 100 U/ml penicillin and 100 μl/ml streptomycin. The cells were cultured at 5% CO_2_ and 37 ˚C in a humidified incubator and treated with or without LPS (1 μg/ml LPS for 6, 24, or 72 h) and Nig for 30 min. After stimulation for the indicated duration, the cells were collected for Western blot analysis, flow cytometry analysis, laser scanning confocal microscopy (Leica, Mannheim, Germany), and transmission electron microscopy.

### Flow cytometric analysis

Experimental and control cells were analyzed using simultaneous FAM-FLICA-CASP-1 and 7AAD staining, and a negative control was included. After CLP or treatment with LPS and Nig, cells were collected and washed three times with cold PBS, resuspended in 290 μl PBS, and then added to 10 μl 30 × FLICA solution. After the cells were incubated in the dark at 37 ℃ for 60 min, 2 ml 1 × apoptosis wash buffer was added, and the cells were mixed and centrifuged at 1500 rpm for 5 min. The cells were resuspended in 100 μl binding buffer, to which 5 μl 7-AAD was added. After being incubated in the dark for 15 min at room temperature, the cells were diluted with 200 μl binding buffer and analyzed by flow cytometry within 1 h using a FACScan instrument (BD Biosciences, Mountain View, CA).

### Western blot analysis

Cells (5 × 10^6^) were collected, washed twice with ice-cold PBS and lysed with lysis buffer. After incubation on ice for 30 min, the homogenates were centrifuged at 14,000 rpm for 30 min at 4 ℃ and then boiled at 95 ℃ for 5 min after being mixed with SDS-loading buffer. The samples were separated by 8%–12% SDS-PAGE (Pulilai Co., Beijing, China), transferred to nitrocellulose membranes and blocked with 10% milk in TBST at room temperature for 2 h. Specific antibodies were used to determine the expression of SESN2 (ab178518, 1:1000), CASP-1 (ab179515, 1:1000), NLRP3 (AG-20B-0014, 1:800), ASC (#67,824, 1:500), GSDMD (ab209845, 1:1000), GRP78 (1:1000), p-PERK (#3179, 1:500), PERK (1:800), ATF4 (ab184909, 1:800), and CHOP (ab11419, 1:1000). A monoclonal anti-β-actin antibody (1:1000) was used as a control for protein loading. Immunoreactivity was visualized by ECL detection system (Amersham Biosciences, Uppsala, Sweden). Protein levels were quantified by densitometric analysis.

### Laser scanning confocal microscopy

Following activation of DCs, the cells were collected, washed with PBS for three times, fixed with 4% paraformaldehyde for 20 min, permeabilized with 0.1% Triton X-100 for 30 min at room temperature, and then blocked with 1% bovine serum albumin (BSA) in distilled water for 30 min. The cells were incubated overnight with a mouse anti-mouse NLRP3 antibody at a 1:200 dilution, an ASC (D2W8U) rabbit monoclonal antibody (specific for mouse) (Alexa Fluor 647 conjugated) at a 1:50 dilution, and an anti-SESN2 antibody at a 1:200 dilution. After being washed with PBS three times, the cells were stained with secondary antibodies, including Alexa Fluor 488-conjugated anti-rabbit and Alexa Fluor 594-conjugated anti-mouse antibodies. The probe ER-Tracker Red (1:1000) was used to selectively stain the ER of live cells for 1 h at 37 °C. The cells were incubated with a FAM-FLICA-CASP-1 probe for 1 h at 37 °C and then with reagents from a death detection kit and TMR red for 1 h at 37 °C. The cells were washed with PBS three times, and then the nuclei were stained with 4’, 6-diamidino-2-phenylindole (DAPI). The cells were observed using a laser scanning confocal microscope (Leica, Mannheim, Germany).

### Transmission electron microscopy

Stimulated cells were fixed in 2.5% glutaraldehyde after experimental manipulations. The cells were photographed using a JEOL JEM 1210 transmission electron microscope (JEOL, Peabody, MA) at 80 or 60 kV on electron microscope film (ESTAR thick base; Kodak, Rochester, NY).

### ELISA

The levels of cytokines (IL-1β, IL-6, IL-10, IL-18, IL-25, HMGB1, and TNF-α) in plasma and supernatants were measured by enzyme-linked immunosorbent assay (ELISA) kits from Abcam, R&D Systems, and RayBiotech following the manufacturers’ indications. The plates were read with a microplate reader (Spectra MR, Dynex, Richfield, MN).

### Statistical analysis

All results are presented as the mean ± standard deviation (SD) of more than three independent experiments. One-way analysis of variance (ANOVA) was used to analyze the significance differences among the groups, and Student’s t-test was used to assess the significance of differences between groups. The survival rate of septic mice was determined by constructing Kaplan–Meier survival curves and using the log-rank-test. *P* < 0.05 was considered statistically significant.

## Supplementary Information

Below is the link to the electronic supplementary material.

Supplementary file1 (DOCX 9485 kb)

## Data Availability

The data that support the findings of this study are available from the corresponding author on reasonable request.
