# Supplementary material for: Sestrin2 protects against lethal sepsis by suppressing the pyroptosis of dendritic cells
Source: Cell Mol Life Sci. 2021 Nov 6;78(24):8209–27. doi: 10.1007/s00018-021-03970-z (PMC8629895; doi:10.1007/s00018-021-03970-z)
Supplement: Supplementary file 1 — Supplementary file1 (DOCX 9485 kb) [file 18_2021_3970_MOESM1_ESM.docx]

**Figure S1-9**

**
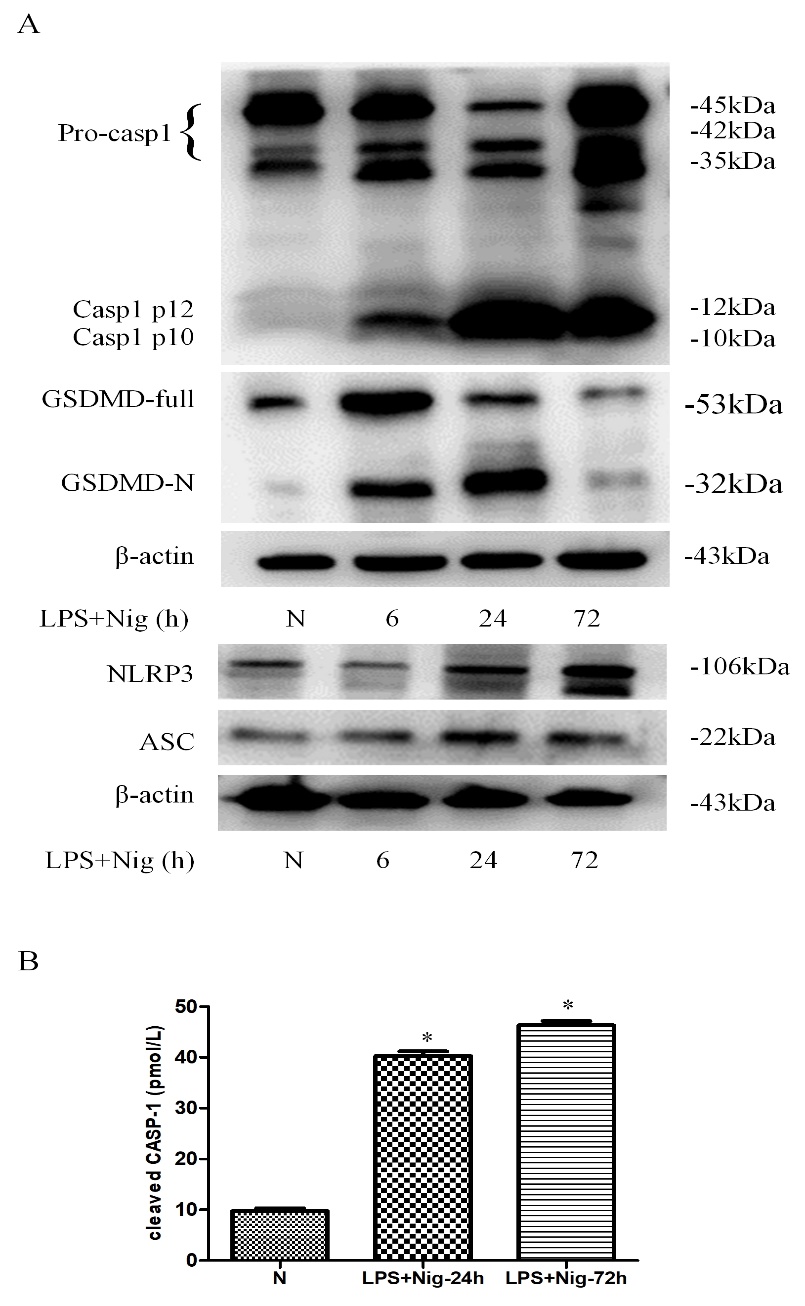
**

**FIG S1. Sepsis induces CASP-1 activation in splenic DCs *in vitro*.** DCs were isolated from the spleen, and primed with 1 μg/ml LPS (6 h, 24 h, or 72 h) and then treated with 20 μM Nig for 30 min. A, Western blotting was used to measure the protein expression of NLRP3, CASP-1, ASC, and GSDMD. The values are protein levels relative to the β-Actin level. The data are from three independent experiments. B, The levels of cleaved CASP-1 in the supernatants of DCs were measured by ELISA. The data are expressed as the mean ± SD (n=5). Statistical significance: ^*^*P<*0.05 versus the control group.

**
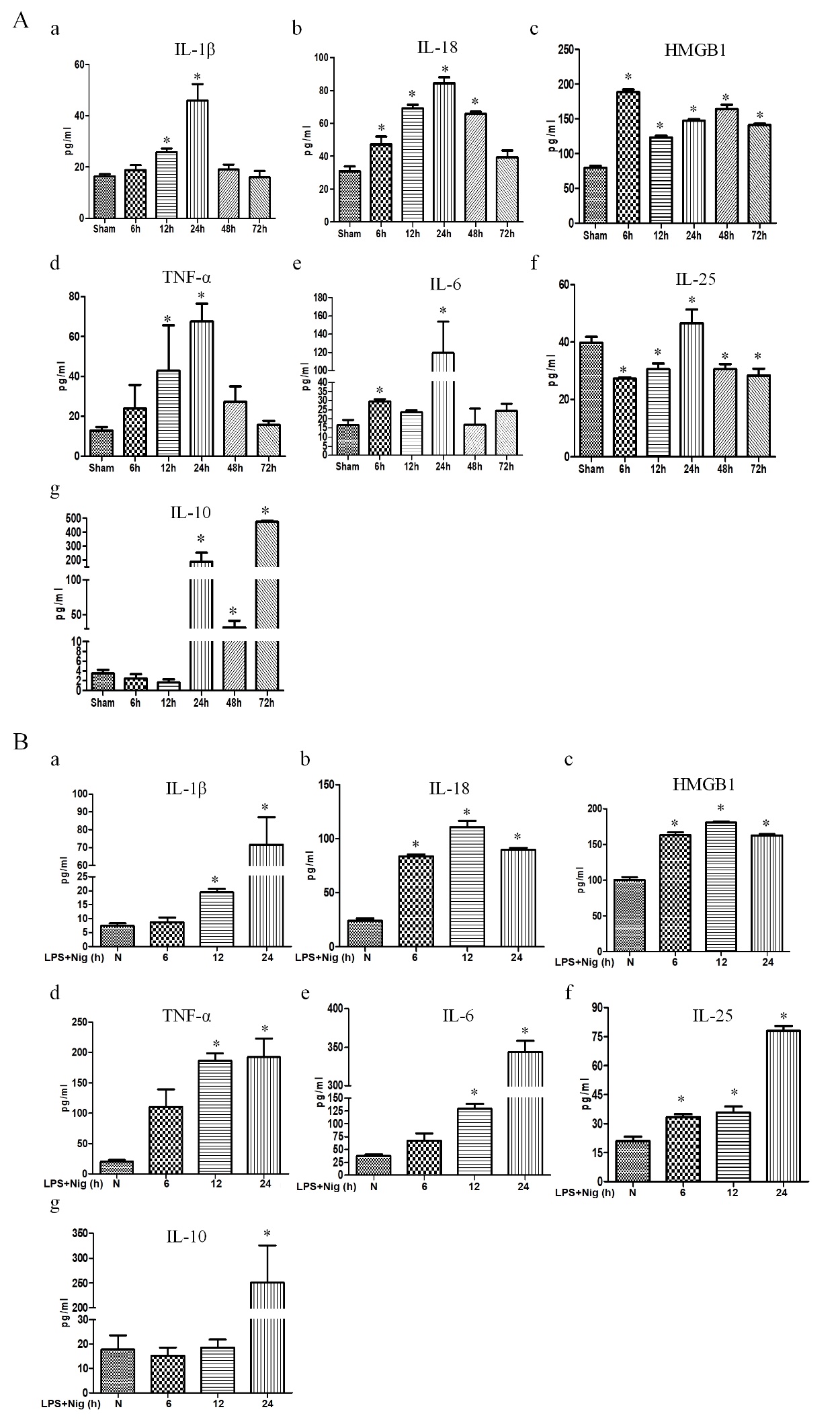
**

**FIG S2. The proinflammatory microenvironment in the context of septic challenge.** A (a-g), The plasma levels of cytokines, including IL-1β, IL-6, IL-10, IL-18, IL-25, HMGB1, and TNF-α, were measured by ELISA at 6 h, 12 h, 24 h, 48 h, and 72 h after CLP or sham operation. B (a-g), The concentrations of IL-1β, IL-6, IL-10, IL-18, IL-25, HMGB1, and TNF-α in the supernatants of DCs were examined by ELISA after treatment with 1 μg/ml LPS (6 h, 12 h, and 24 h) followed by 20 μM Nig for 30 min. The data are expressed as the mean ± SD (n=4–6). ^*^*P<*0.05 versus the sham or normal group.


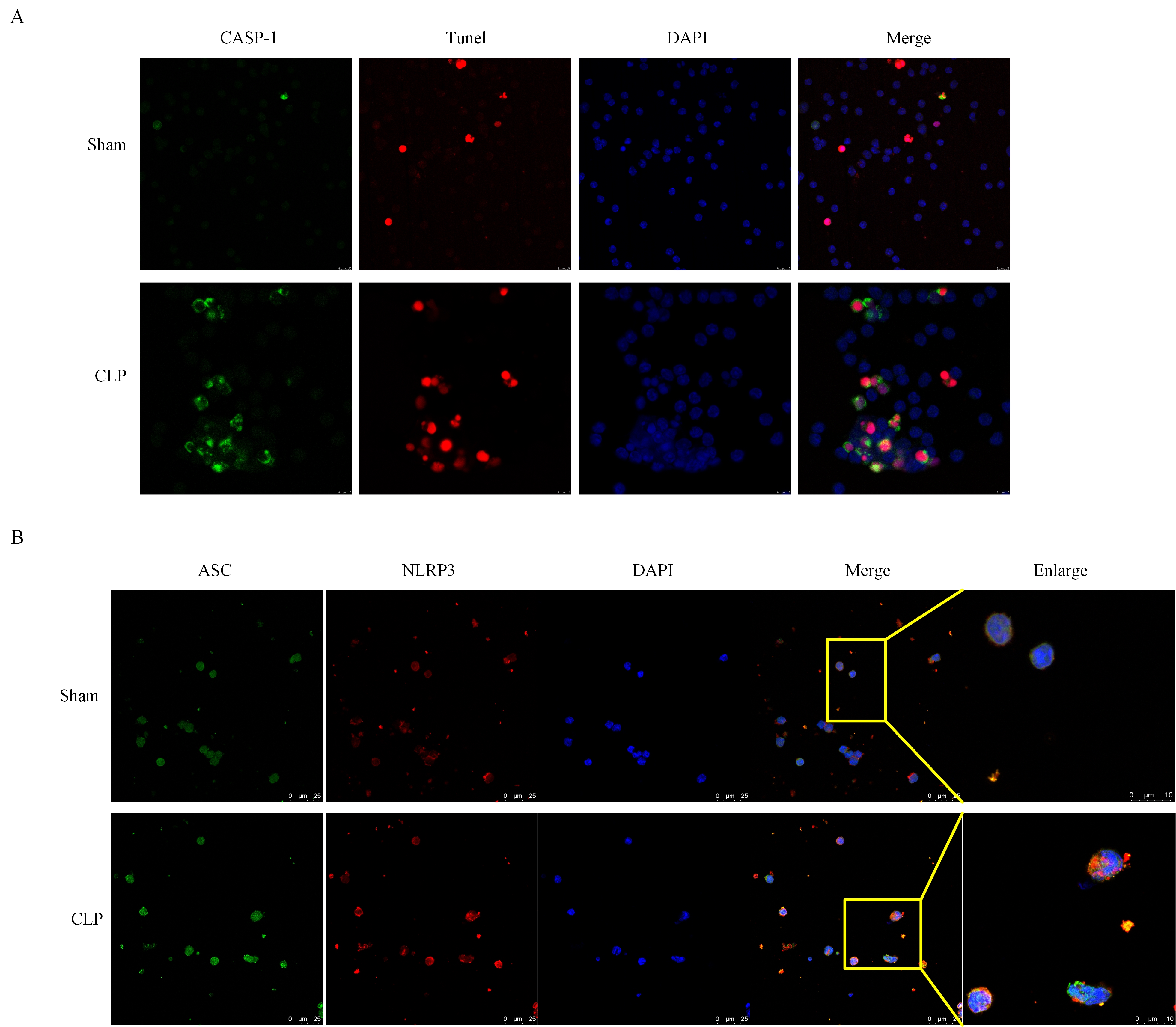


**FIG S3. Splenic DCs express NLRP3 inflammasome components.** Splenic DCs were isolated from mice at 24 h after CLP or sham operation. A, Representative confocal immunofluorescence images of CASP-1 and TUNEL staining in primary splenic DCs. FAM-FLICA-labeled CASP-1 protein, red TUNEL staining, and DAPI (blue)-stained nuclei are shown. B, Representative confocal immunofluorescence images of NLRP3 colocalized with ASC in primary splenic DCs. DyLight 488 (green)-labeled ASC protein, DyLight 594 (red)-labeled NLRP3 protein, and DAPI (blue)-stained nuclei are shown. The data shown are representative of 3 independent experiments.


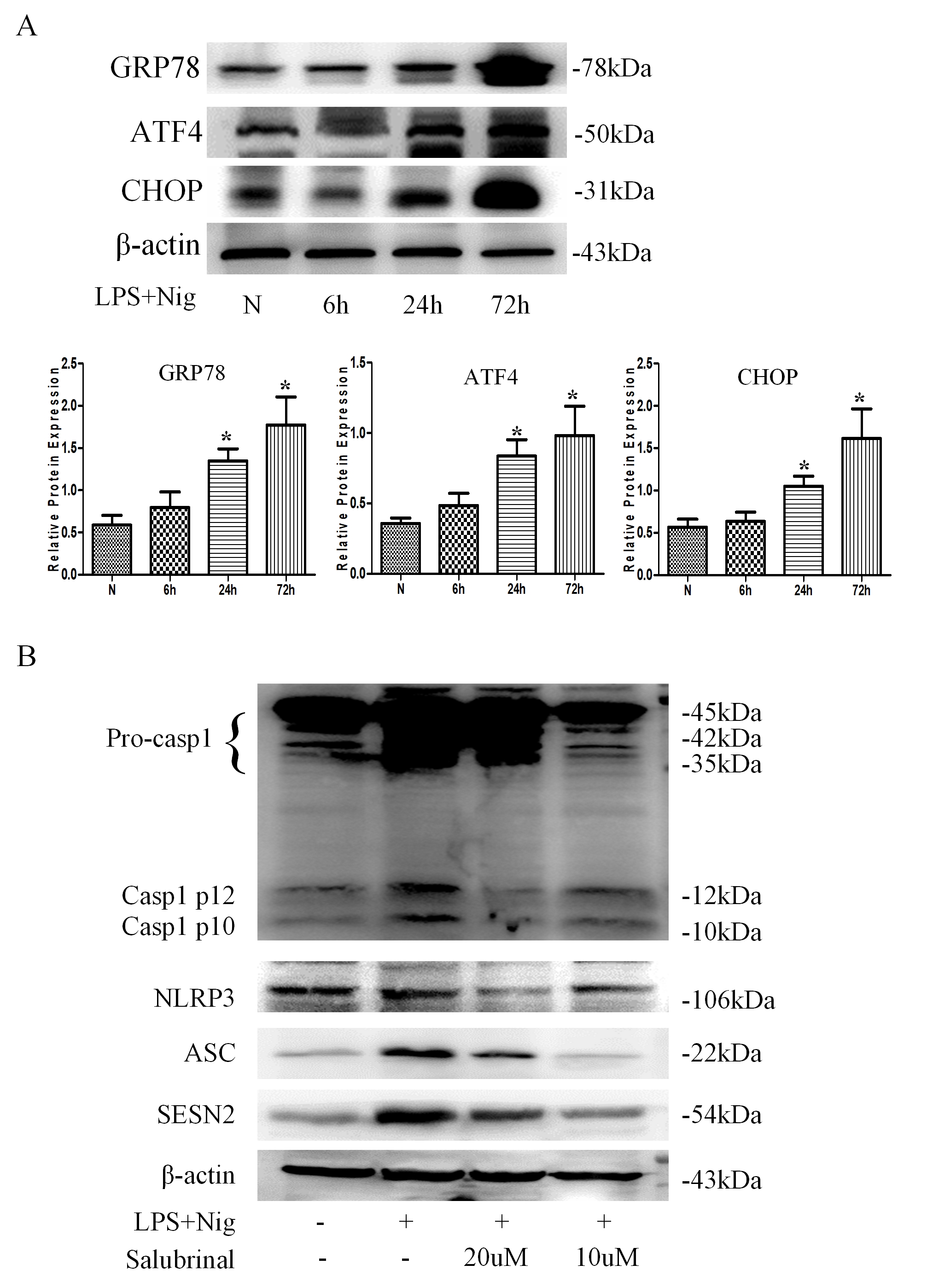


**FIG S4. ERS participates in NLRP3/CASP-1-dependent pyroptosis and contributes to SESN2 expression in DCs *in vitro*.** A, Splenic DCs were primed with 1 μg/ml LPS (6 h, 24 h, or 72 h) and then treated with 20 μM Nig for 30 min. The expression of ERS markers (GRP78, ATF4, and CHOP) was analyzed by Western blotting. B, Cells were pretreated with various dosages of salubrinal for 1 h, stimulated with 1 μg/ml LPS for 24 h and then treated with 20 μM Nig for 30 min. CASP-1, NLRP3, ASC, and SESN2 expression in DCs was assessed by Western blotting. β-Actin served as an internal control. The data shown are representative of 3 independent experiments. Statistical significance: ^*^*P<*0.05 versus the control group.


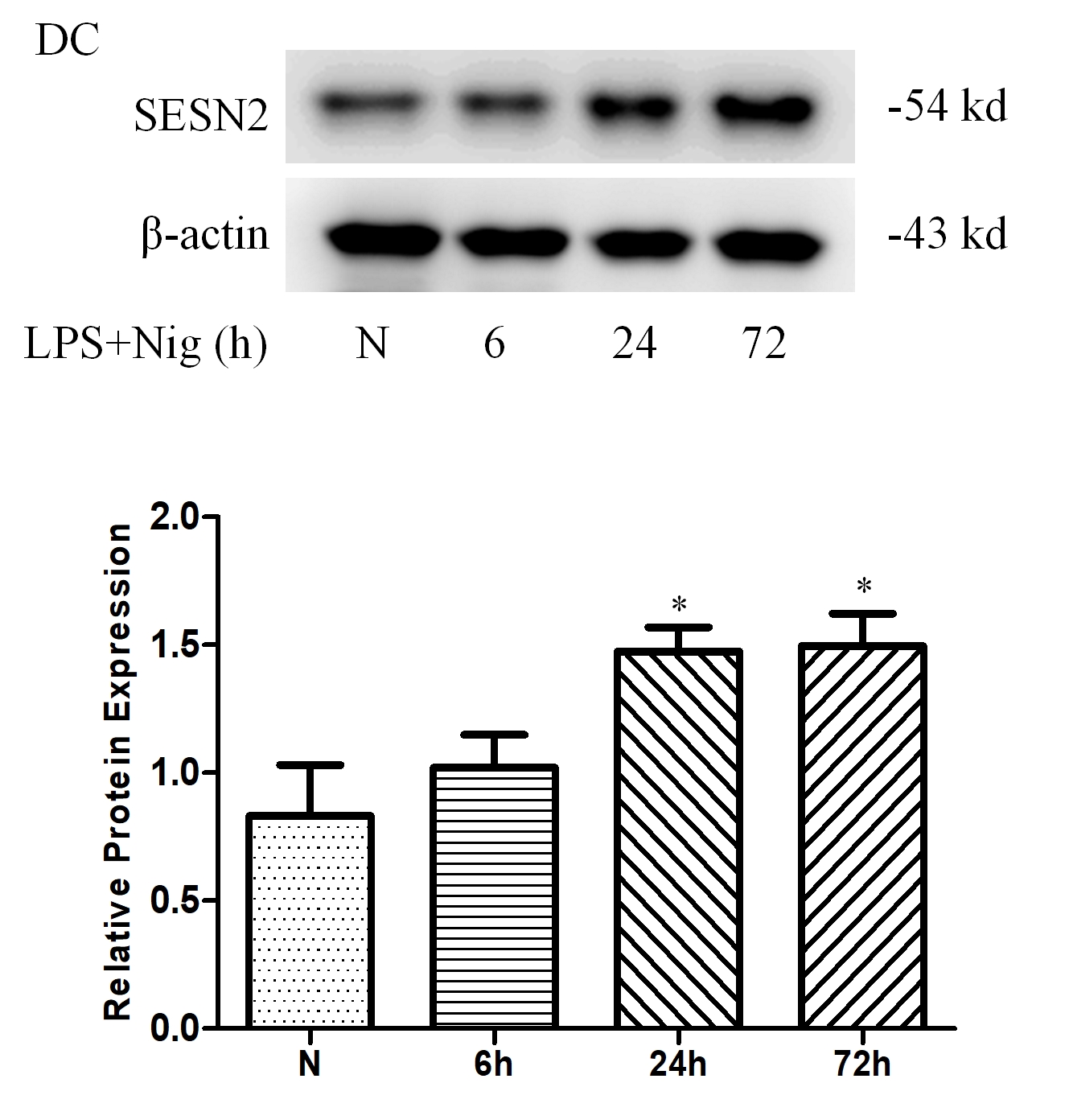


**FIG S5. SESN2 expression in DCs *in vitro***. The protein expression of SESN2 in DCs primed with 1 μg/ml LPS for 6 h, 24 h, or 72 h, and then treated with Nig for 30 min was evaluated by Western blotting. β-Actin served as an internal control. The data are presented as the mean ± SD of three independent experiments. Statistical significance: ^*^*P<*0.05 versus the control group.


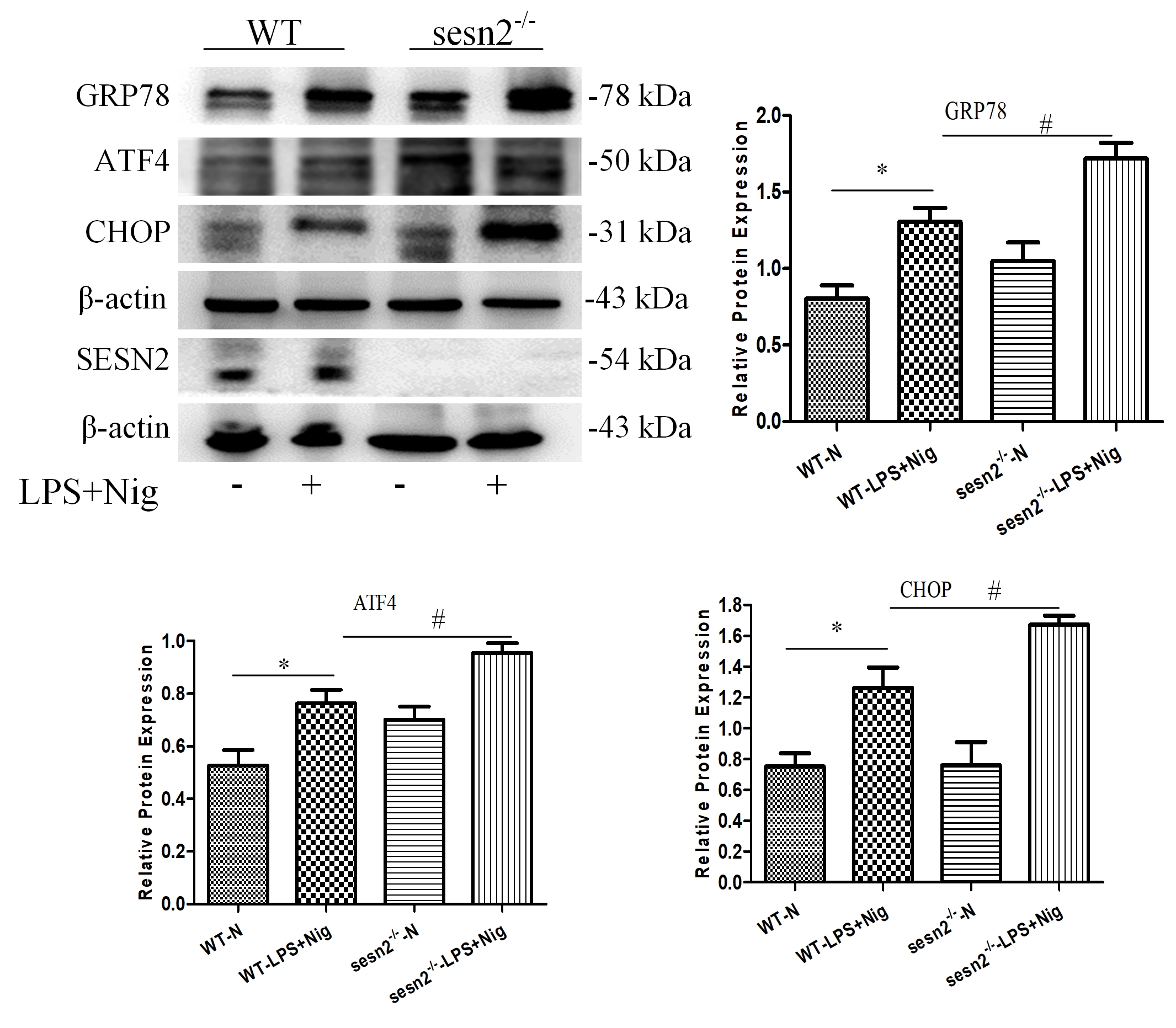


**FIG S6. SESN2 deficiency aggravates ERS *in vitro*.** Western blotting was used to measure the protein expression of GRP78, ATF4, and CHOP in DCs from WT and SESN2^-/-^ mice after treatment with 1 μg/ml LPS for 24 h followed by Nig (20 μM, 30 min). The data are presented as the mean ± SD of three independent experiments. Statistical significance: ^*^*P<*0.05 versus the control group.


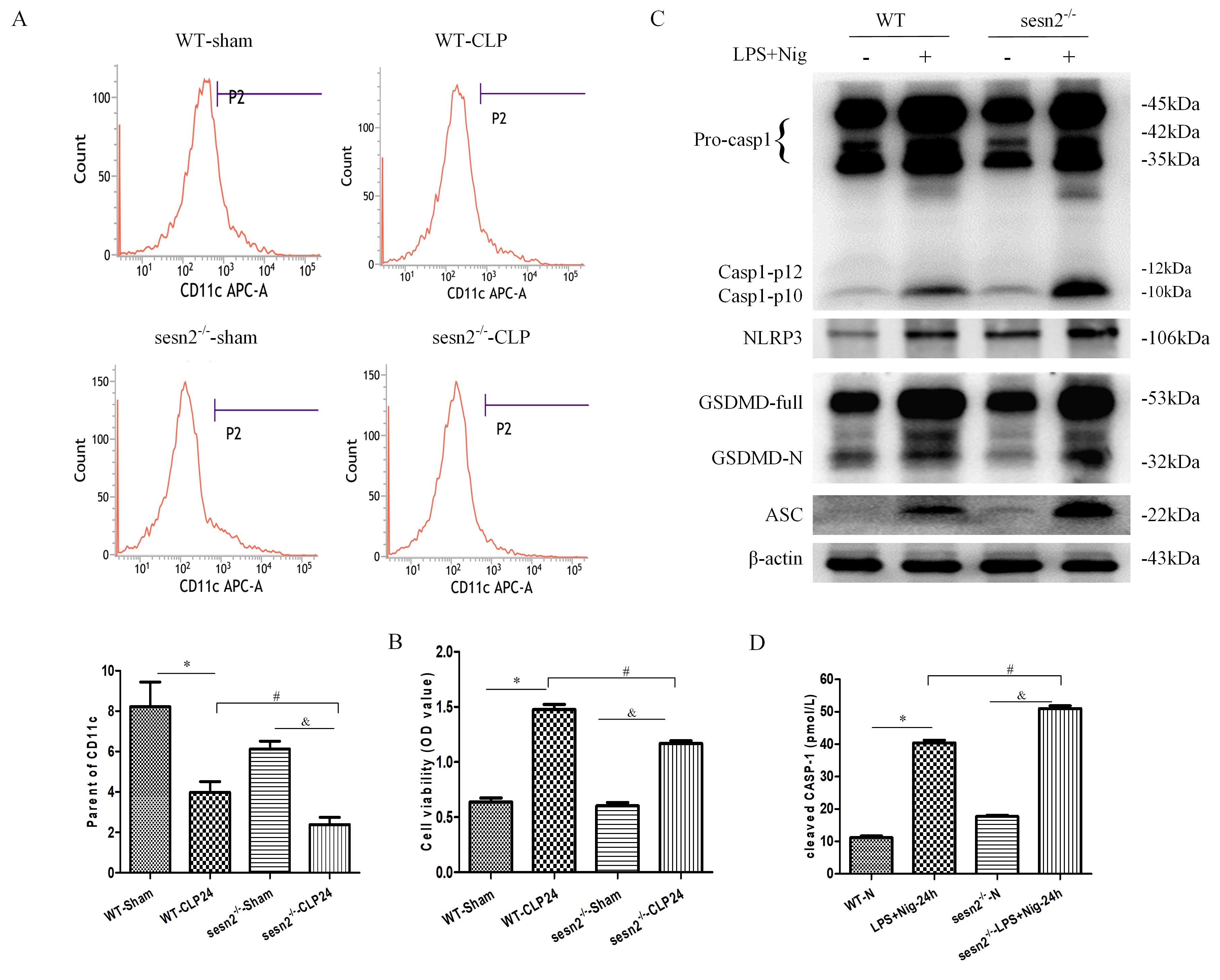


**FIG S7. Genetic deficiency of SESN2 exacerbates the decrease in DC number and NLRP3/CASP-1 hyperactivation.** DCs were isolated from the spleens of WT and SESN2^-/-^ mice at 24 h following CLP. A, The percentage of CD11c^+^ cells among splenic lymphocytes was examined by flow cytometry. Representative flow cytometry plots are shown on the left (n=5). B, DC viability was examined with a CCK-8 kit (n=10). C, The expression of CASP-1, GSDMD, NLRP3, and ASC in DCs from WT and SESN2^-/-^ mice primed with 1 μg/ml LPS for 24 h followed by treatment with 20 μM Nig for 30 min was analyzed by immunoblotting. β-Actin served as an internal control. D, Cleaved CASP-1 levels in the superantants of DCs priming with 1 μg/ml LPS for 24 h and then treated with 20 μM Nig for 30 min were measured by ELISA. The data are presented as the mean ± SD. Statistical significance: ^*^*P<*0.05 versus the control group.


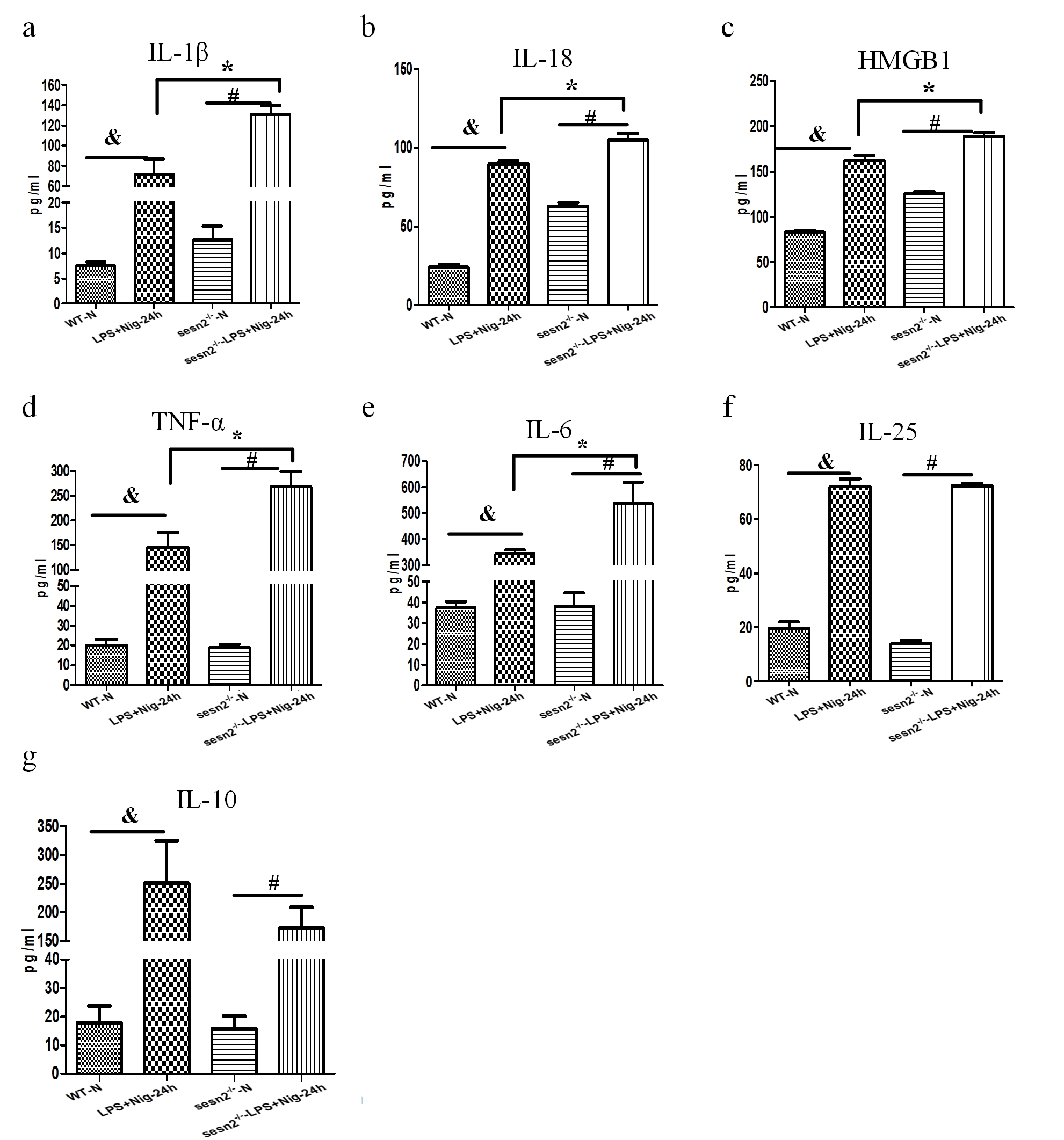


**FIG S8. Knockout of SESN2 augments the inflammatory response.** DCs from the spleens of WT and sesn2^-/-^ mice were primed with 1 μg/ml LPS for 24 h and then treated with 20 μM Nig for 30 min. IL-1β, IL-6, IL-10, IL-18, IL-25, HMGB1, and TNF-α concentrations in the supernatants of DCs were assayed by ELISA (n=6). The data are presented as the mean ± SD. Statistical significance: ^*^*P*<0.05 versus the control group.


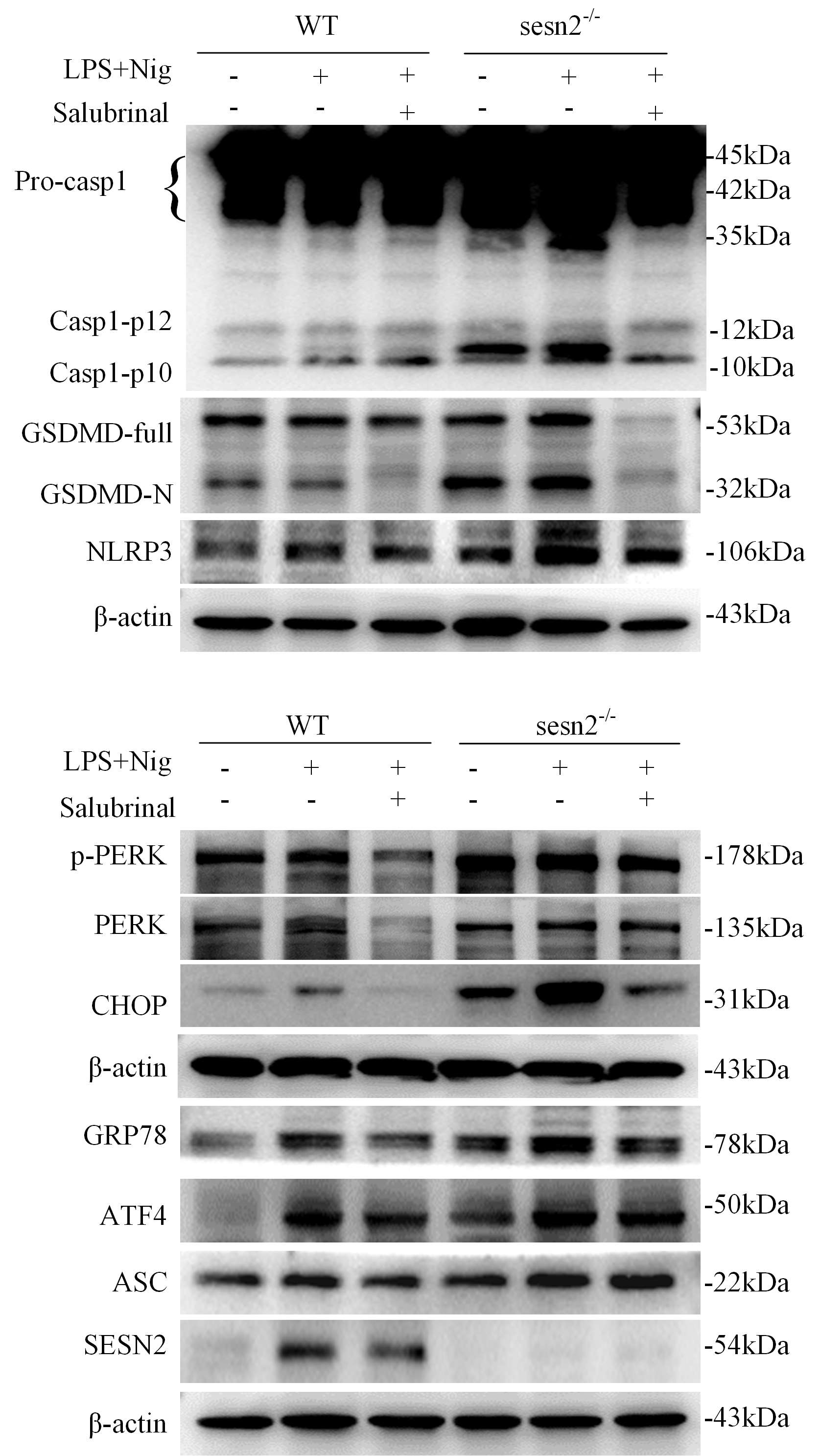


**FIG S9. The PERK-ATF4-CHOP pathway is critically involved in the inhibitory effect of SESN2 on DC pyroptosis.** DCs were isolated from the spleen, primed with 1 μg/ml LPS for 24 h and treated with 20 μM Nig treatment for 30 min or with Nig and salubrinal. Immunoblot analysis of the expression of inflammasome markers, including CASP-1, GSDMD, NLRP3, ASC and signaling molecules, such as PERK, ATF4, CHOP, and GRP78, was performed. The data shown are representative of 2 independent experiments.
